# Design of a single inductor bidirectional DC converter for V2V energy transfer: On board charger architecture

**DOI:** 10.1038/s41598-026-57506-1

**Published:** 2026-06-30

**Authors:** Hazem H. Mostafa, Amr M. Ibrahim, Fathy Z. Amer, Eman F. Sawires

**Affiliations:** 1 Energy and Renewable Energy Department, Faculty of Engineering, Egyptian Chinese University, Cairo, Egypt; 2 Electrical Power and Machines Department, Faculty of Engineering, Ain Shams University Cairo, Cairo, Egypt; 3 Electronics and Communications Engineering Department, Faculty of Engineering, Capital University (formerly Helwan University), Cairo, Egypt

**Keywords:** Bi-directional dc converter, Multi-port, Wireless power transfer, G2V, V2V, Energy science and technology, Engineering

## Abstract

This paper presents a Single-Inductor Bidirectional Converter (SIBC) for unified onboard Electric Vehicle (EV) charging, integrating grid, battery, and wireless ports within a single power stage. The topology enables native Grid-to-Vehicle (G2V), Wireless-to-Vehicle (W2V), and Vehicle-to-Wireless (V2W) operation without hardware reconfiguration, eliminating cascaded converter–inverter structures. Non-ideal steady-state and small-signal models are developed, revealing mode-dependent dynamics including a right-half-plane zero in V2W mode that constrains bandwidth. A two-loop Average Current-Mode Control (ACMC) is proposed to mitigate this limitation, achieving 40× bandwidth improvement over conventional voltage-mode control. Parametric sensitivity analysis of inductor equivalent series resistance establishes quantitative design boundaries for sustaining conversion efficiency above 90%. Scalability assessment to 3 kW operation demonstrates compatibility with silicon carbide devices and a bridgeless totem-pole PFC front-end achieving THD less than 4% and power factor higher than 0.99, satisfying IEC 61000-3-2 Class A with higher than 35 dB ripple rejection at the battery terminals. Experimental validation using a 136 W prototype achieves 93.4% transmitter and 95.07% receiver DC–DC efficiency, with an overall end-to-end efficiency of 75.03% including an 84.5% wireless link. A 1 kW interim hardware test confirms scalable operation, while simulation validates CC-CV battery charging compatibility. The results demonstrate that the SIBC architecture is not the dominant source of system losses and provides a compact, scalable foundation for advanced bidirectional EV charging systems.

##  Introduction

The transportation sector accounts for nearly 24% of direct CO₂ emissions from fuel combustion, making vehicle electrification a critical mitigation strategy^1,2^. Global EV sales exceeded 14 million units in 2023 and are projected to constitute 60% of new vehicle sales by 2030 ^3^. Yet this rapid adoption exposes critical bottlenecks: high purchase costs, limited battery capacity, grid overload risks from uncoordinated charging, and inadequate charging infrastructure density particularly in urban areas where 40% of households lack dedicated parking^4^. Current infrastructure growth rates lag behind EV adoption, undermining range confidence and consumer acceptance^3,4^.

Instead of relying exclusively on fixed charging infrastructure, which depends on grid availability and predefined charging locations, Vehicle-to-Vehicle (V2V) charging provides a flexible alternative that enables direct energy transfer between electric vehicles on demand^5–7^. This decentralized approach enhances operational resilience, supports emergency range extension, and enables dynamic energy sharing without requiring immediate access to stationary charging stations. An EV can extend its driving range by approximately 50 km after receiving nearly 10 kWh from another vehicle, effectively mitigating range anxiety without infrastructure dependency^6^. Beyond emergency scenarios, V2V systems enable cooperative charging among fleet vehicles, peak demand energy balancing, and participation in Vehicle to Grid services, thereby improving operational flexibility and system reliability^7^. The underlying framework for V2V operation requires real time coordination of parameters such as State of Charge, State of Health, battery chemistry compatibility, and estimated travel range to ensure safe and efficient energy exchange^8–10^. However, most reported studies focus primarily on conductive wired architectures, while wireless V2V implementation remains relatively unexplored.

V2V power transfer can be implemented through conductive (plug-in) or wireless power transfer (WPT) approaches. Conductive charging is technologically mature and achieves high efficiency; however, it introduces mechanical wear, connector limitations, and user-handling constraints^11–14^. WPT enables contactless energy exchange and improved flexibility, but its performance depends strongly on magnetic coupling and coil alignment^14,15^. Coil optimization has addressed these limitations, with conventional rectangular coils achieving 82–87% efficiency under controlled alignment and advanced bipolar configurations reaching up to 88% with misalignment^15^. Yet these approaches focus on magnetic coupling rather than the power electronics challenge: practical WPT systems typically cascade a DC–DC converter with a dedicated high-frequency inverter and compensation network^12,15^, increasing component count and complicating bidirectional control. Moreover, recent three-port converter solutions employ high-frequency transformers with H-bridge inverters^12,14^, achieving multi-port energy management but requiring complex magnetic structures and auxiliary switches^12^.

Existing EV charging research generally follows two parallel directions. The first focuses on high-efficiency bidirectional DC–DC converters for Grid-to-Vehicle (G2V) and Vehicle-to-Grid (V2G) applications, emphasizing soft-switching techniques, multiport integration, and high conversion efficiency, with reported efficiencies exceeding 93–98%^4,8,12^. In parallel, WPT systems concentrate on magnetic coupling optimization, coil geometry, and compensation network design to improve transfer efficiency and misalignment tolerance^16–22^. Despite this progress, practical implementations typically realize wireless capability by cascading a DC–DC converter with a dedicated high-frequency inverter and resonant compensation network ^11,13,17^. Such cascaded architectures increase component count, introduce duplicated energy-processing stages, and complicate bidirectional control. Moreover, most reported multiport converters are designed exclusively for wired operation and do not inherently integrate a wireless excitation port within the same power stage^4,18,19,23^. Recent three-port wireless charging systems achieve multi-source integration using transformer-coupled H-bridge topologies^12,14,15^, but these require complex magnetic structures, multiple active bridges, and mode-dependent switching strategies that increase system volume and control complexity. Consequently, bidirectional DC–DC conversion and wireless interfacing are commonly treated as separate subsystems rather than a unified power-processing architecture. Few reported solutions demonstrate native integration of bidirectional regulation and high-frequency wireless excitation within a single compact topology particularly one that eliminates magnetic transformers and operates through unified modulation without hardware reconfiguration.

To address this structural limitation, this work proposes a Single-Inductor Bidirectional Converter (SIBC) in which the wireless interface is embedded directly within the fundamental power conversion stage. Unlike transformer-coupled multiport converters^12,14^ or reconfigurable systems requiring mode-switching relays^24–26^, the proposed SIBC achieves native G2V, Wireless-to-Vehicle (W2V), and Vehicle-to-Wireless (V2W) operation using a unified modulation framework and a single energy storage element, eliminating redundant conversion layers, magnetic transformers, and hardware reconfiguration while preserving high intrinsic DC–DC efficiency.

A comprehensive non-ideal steady-state analysis incorporating parasitic resistances is derived to quantify the influence of inductor Equivalent Series Resistance (ESR) on voltage gain and efficiency across all operating modes. This analysis establishes quantitative magnetic design boundaries to sustain conversion efficiency above 90% providing actionable criteria for inductor selection that are validated through parametric simulation. Additionally, a complete small-signal model is developed to characterize the dynamic behavior of the converter in both step-down and step-up modes, confirming the presence of a boost-type Right-Half-Plane (RHP) zero in V2W operation that fundamentally constrains voltage-loop bandwidth. To circumvent this limitation, a two-loop Average Current-Mode Control (ACMC) is proposed, enhancing the bandwidths and Phase Margins (PM). This yields an improvement over voltage-mode control while maintaining robust stability and low transient response essential for EV charging. To bridge the gap between proof-of-concept and practical deployment, this manuscript further validates the SIBC through CC–CV battery charging simulation, WPT misalignment analysis, a complete grid-side PFC front-end with IEC 61000-3-2 Class A harmonic compliance, a 1 kW interim hardware scalability test, and an extended multi-metric performance comparison including power density, component count, and semiconductor stress. Scalability assessment demonstrates that the SIBC architecture is compatible with silicon carbide (SiC) wide-bandgap devices, enabling 100 kHz switching frequencies that reduce magnetic volume by 5x and achieve high semiconductor efficiency without topological modification. Thermal analysis confirms practical feasibility with heat sink under natural convection at 3 kW, with clear pathways to 6.6 kW Level 2 onboard charger applications through forced-air or liquid-cold-plate thermal management.

1. A compact SIBC architecture integrating grid, battery, and wireless ports within a single power stage without magnetic transformers or auxiliary switches, distinguishing from transformer-coupled multiport converters.
2. Native bidirectional wired/wireless operation: Seamless G2V, W2V, and V2W functionality achieved through unified modulation without hardware reconfiguration or mode-switching relays, eliminating the structural switching required by existing reconfigurable systems.
3. Non-Ideal steady-state and small-signal modeling: Closed-form gain equations including parasitic effects revealing inductor ESR impact on efficiency, with quantified design boundaries; and a complete small-signal model identifying the RHP zero constraint in V2W mode.
4. Advanced control architecture: A two-loop ACMC solution achieving bandwidth improvement over voltage-mode control, with validated closed-loop dynamic response suitable for Power Factor Correction (PFC) ripple rejection and battery transient management.
5. Experimental validation, sensitivity analysis, and scalability assessment: Hardware prototype validates bidirectional wireless operation; parametric sensitivity confirms analytical predictions; 3 kW scalability quantified via SiC device and thermal analysis.

## The proposed converter topology

**Fig. 1:**
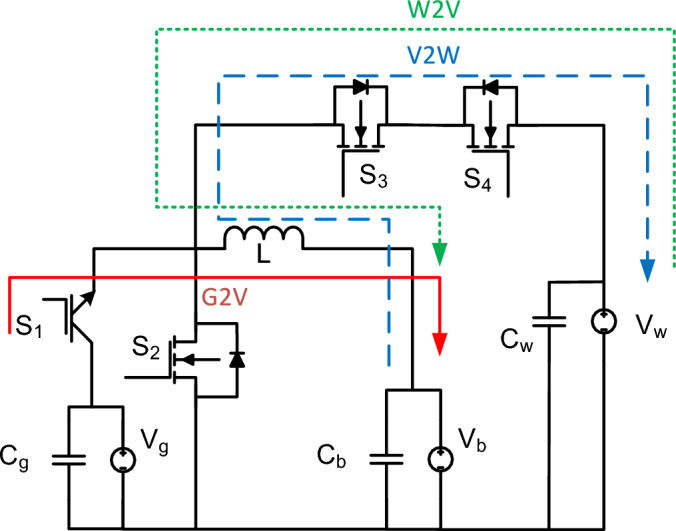
SIBC on board converter.

**Fig. 2:**
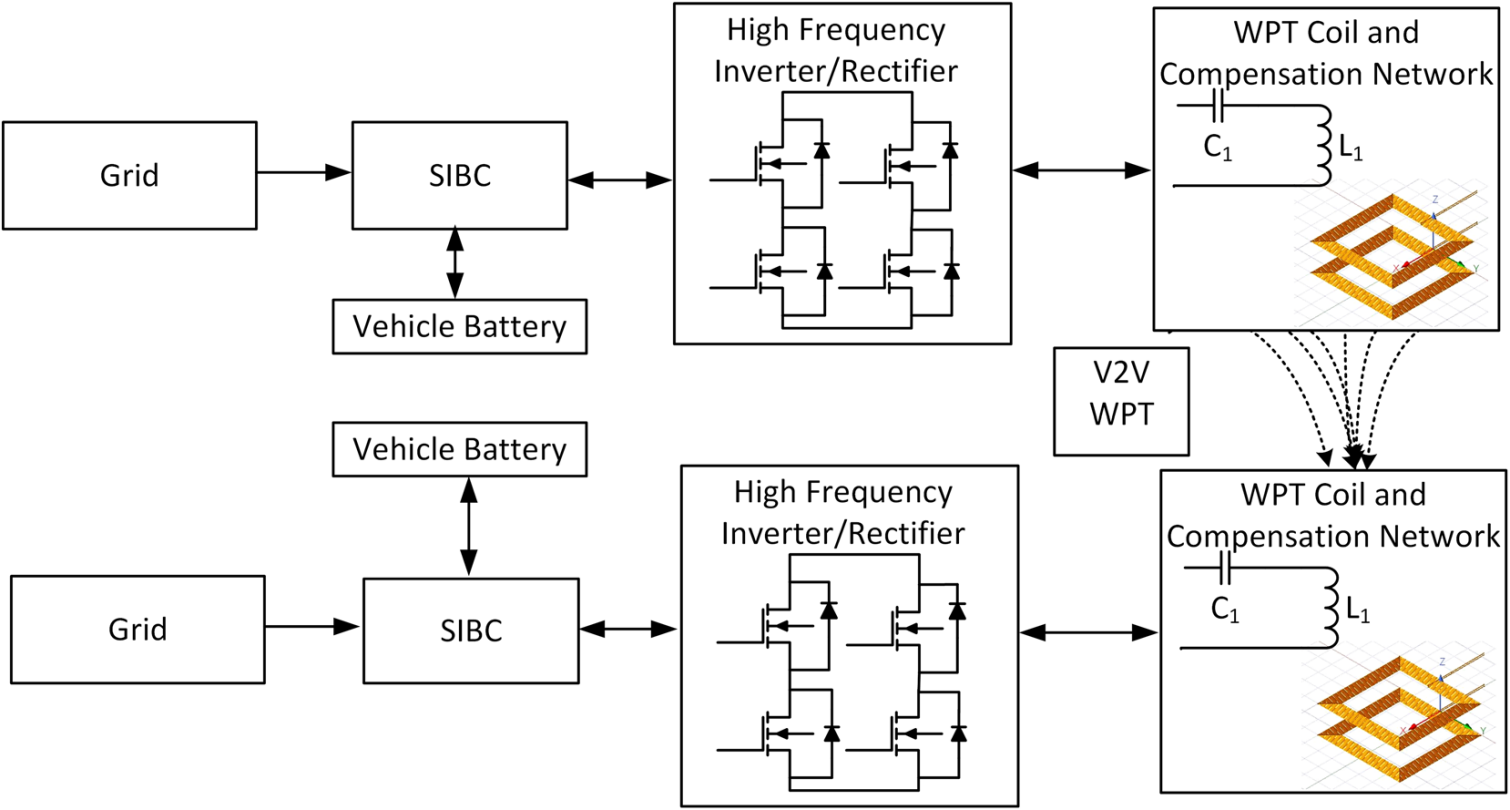
Block diagram of the on board charging system.

The proposed SIBC is a multiport converter and is specifically designed to support G2V and V2V charging operations with high efficiency and flexible control. As illustrated in Fig. 1, the converter integrates high voltage gain and bidirectional power transfer capabilities. The topology includes four switches (S₁–S_4_), one inductor (L), and three filter capacitors (C_W_, C_g_, C_b_) connected across three active ports: the grid port (V_g_), the wireless power transfer port (V_W_), and the vehicle battery port (V_b_). During G2V mode, the converter regulates the energy flow from the grid to the vehicle battery through, maintaining stable voltage and minimizing conversion losses. In V2V mode, the direction of power flow reverses, allowing one vehicle’s battery to charge another wirelessly via the same converter configuration. The incorporation of a high-frequency inverter within the WPT section enhances power density and ensures efficient resonant energy coupling between vehicles. Operating in Continuous Conduction Mode (CCM) further reduces current ripple and switching stress, ensuring smooth transitions between G2V and V2V modes. Overall, the SIBC topology offers compact design, high gain, and reliable bidirectional functionality, demonstrating suitability for integrated wireless EV charging applications. The SIBC with integrated high frequency inverter and WPT coil block diagram is shown in Fig. 2.

The integration of the WPT interface and high-frequency inverter stage extends the converter’s functionality to support both G2V and V2V operation without modifying the fundamental voltage conversion relationships. The SIBC operates in three distinct modes corresponding to different energy transfer scenarios. In G2V mode, it functions as a step-down converter, regulating grid power to charge the vehicle battery. In W2V mode, the converter similarly operates in a step-down configuration to process power received through the wireless port and deliver controlled charging to the battery. In V2W mode, the topology transitions to step-up operation, boosting the battery voltage to supply the wireless interface for power transfer. This multi-mode capability enables seamless bidirectional energy exchange across wired and wireless ports within a single architecture.

### First mode (G2V)

**Fig. 3:**
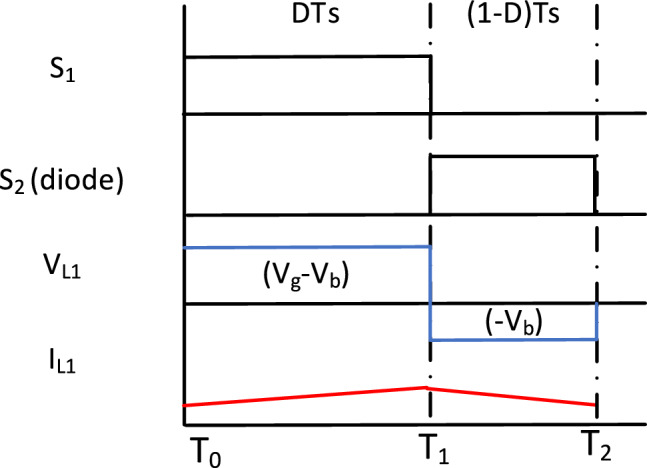
Wave forms of SIBC operation in G2V mode.

**Fig. 4:**
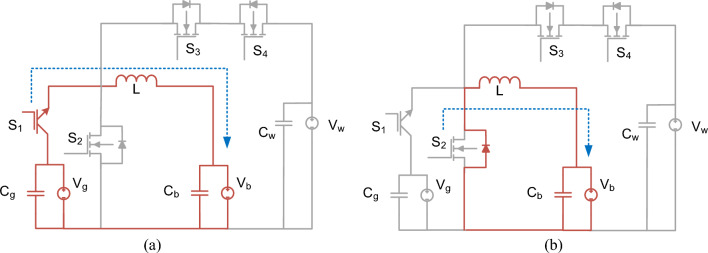
SIBC converter operation in G2V mode (**a**) Interval 1; (**b**) Interval 2.

In step-down operation, the waveforms can be inferred as presented in Fig. 3. At the first time interval, T_0_ to T_1_: S_1_ is on, and the rest of the switches are off. The grid voltage charges L. The circuit of SIBC and its operation is shown in Fig. 4(a). The following equation (1) is the expression for the voltages of the inductor L.

1 $${V}_{L}={V}_{g}-{V}_{b}$$

As for the second time interval T_1_ to T_2_: current flows through S_2_ (diode) to V_b_ while the rest of the switches are off. The battery is supplied by a discharge current from L as shown in Fig. 4(b). Voltage of inductor L is expressed as follows:

2 $${V}_{L}=-{V}_{b}$$

The gain of SIBC converter in mode 1 (step-down) is obtained as follows:

3 $$\frac{{V}_{b}}{{V}_{g}}=D$$

where D is the duty cycle, V_b_ is the car battery voltage, V_g_ is the grid voltage, V_W_ is the voltage of the WPT system.

###  W2V and V2W modes

**Fig. 5:**
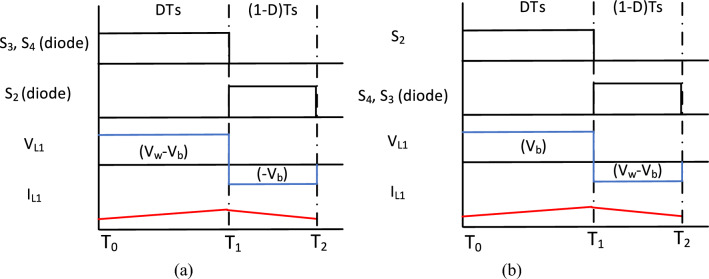
Waveforms of SIBC operation: (**a**) W2V; (**b**) V2W.

**Fig. 6:**
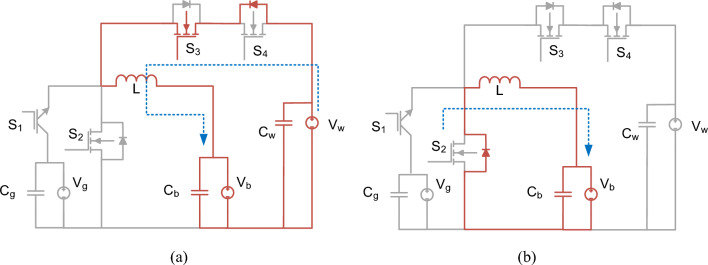
SIBC Converter operation in W2V mode (**a**) Interval 1; (**b**) Interval 2.

The bidirectional operation is achieved by reconfiguring switch control logic. In V2W mode, S_2_, S_4_, and the diode of S_3_ operate as boost-side switches, while S_2_, S_3_, and the diode of S_4_ are gated complementarily for step-down operation in W2V mode. The steady-state waveforms are presented in Fig. 5(a) for W2V mode and Fig. 5(b) for V2W mode. As for the W2V mode, the operation of the circuit is shown in Fig. 6.

Similar to G2V operation, during the first interval, S_3_ and S_4_ diode are on, and the rest of the switches are off. The wireless voltage charges L. The circuit of SIBC and its operation are shown in Fig. 6(a). The expression for the voltages of the inductor L.

4 $${V}_{L}={V}_{w}-{V}_{b}$$

As for the second interval, current flows through S_2_ (diode) to V_b_ while the rest of the switches are off. The battery is supplied by a discharge current from Las shown in Fig. 6(b). Voltage of inductor L is obtained as:

5 $${V}_{L}=-{V}_{b}$$

The gain of SIBC converter in mode 2 (step-down) is obtained as follows:

6 $$\frac{{V}_{b}}{{V}_{w}}=D$$

**Fig. 7:**
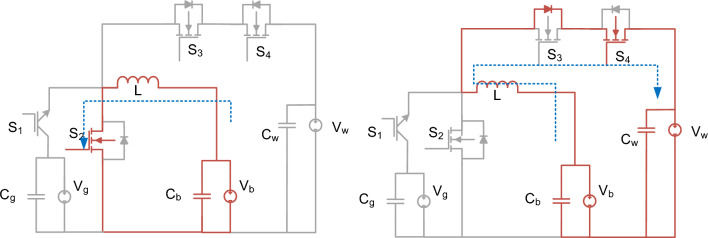
SIBC converter operation in V2W mode (**a**) Interval 1; (**b**) Interval 2.

For the final mode V2W the operation of the circuit is shown in Fig. 7.

At the first interval, S_2_ is on, and the rest of the switches are off. The vehicle battery voltage charges L. The circuit of SIBC and its operation are shown in Fig. 7(a). The following equation (7) is the expression for the voltages of the inductor L.

7 $${V}_{L}={V}_{b}$$

As the second interval, current flows through S_4_ and S_3_ diode to V_W_ while the rest of the switches are off. The wireless port is supplied by a discharge current from L as shown in Fig. 7(b). Voltage of inductor L is expressed as follows:

8 $${V}_{L}={V}_{w}-{V}_{b}$$

The gain of SIBC converter in mode 3 (step-up) is obtained as follows:

9 $$\frac{{V}_{w}}{{V}_{b}}=\frac{1}{1-D}$$

### SDCM for SIBC

The preceding analysis assumes CCM, where the inductor current remains positive throughout the switching cycle. Under light-load conditions, the converter may enter Discontinuous Conduction Mode (DCM), where the inductor current falls to zero during a portion of the switching period. The boundary between CCM and DCM occurs when the minimum inductor current reaches zero. For the V2W boost configuration, the inductor current ripple is^18^:

10 $${\Delta I}_{L}=\frac{{V}_{b}D{T}_{s}}{L}$$

where T_s_ is the switching time. At the boundary, the average inductor current equals half the ripple:

11 $${I}_{o-boundary}=\frac{{V}_{b}D{T}_{s}}{2L(1-D)}$$

Defining the dimensionless parameter K:

12 $$K=\frac{2L}{R{T}_{s}}$$

where R is the load resistance. The converter operates in CCM when K > K_crit_​ and in DCM when K < K_crit_​ , where:

13 $${K}_{crit}=D {(1-D)}^{2}$$

When operating in DCM (K<Kcrit​ ), the voltage gain becomes load-dependent.

14 $$\frac{{V}_{w}}{{V}_{b}}=\frac{1+\sqrt{1+4{D}^{2}/K}}{2}$$

Similarly, the DCM voltage gain under G2V and W2V is:

15 $$\frac{{V}_{b}}{{V}_{g,w}}=\frac{2}{1+\sqrt{1+4K/{D}^{2}}}$$

Where K_crit_ for the G2V and W2V modes is calculated as:

16 $${K}_{crit}=\frac{2D {(1-D)}^{2}}{2-D}$$

## Voltage stress, current stress, and non-ideal analysis of SIBC.

### Voltage stress

The voltage stress across each switching device is obtained for each of the three modes of the SIBC using the circuit analysis in the previous section. The maximum voltage across MOSFETs and IGBTs is illustrated in Table 1^18,19^.

**Table 1:** MOSFETs and IGBTs Voltage Stresses for SIBC.

SIBC	S_1_	S_2_	S_3_	S_4_
	${V}_{g}$	${V}_{g}$	${V}_{w}$	${V}_{w}$

### Current stress

The current stress on MOSFETs and IGBTs in each mode of the SIBC converter can be determined by using the equivalent circuits for each mode. The maximum current across MOSFETs and IGBTs is illustrated in Table 2^18,19^.

**Table 2:** MOSFETs and IGBTs current stresses for SIBC.

SIBC Mode	S_1_	S_2_	S_3_	S_4_
G2V	$D{I}_{g}$	$(1-D){I}_{g}$	-	-
W2V	-	${(1-D)I}_{w}$	$D{I}_{w}$	$D{I}_{w}$
V2W	-	$D{I}_{b}$	$(1-D){I}_{w}$	$(1-D){I}_{w}$

### Non-ideal analysis

To accurately characterize the SIBC performance under realistic operating conditions, this section presents a non-ideal analysis that incorporates key parasitic resistances into the circuit model. Despite the topology’s high efficiency and reduced component count, which inherently minimize loss mechanisms, on-state resistances of the switching devices (r_S_), the inductor’s equivalent series resistance (r_L_), MOSFET body diode forward resistance (r_d_), and R collectively contribute to measurable deviations from ideal behavior. Following the analytical framework established in preceding sections, volt-second balance across inductor L is applied to derive the steady-state voltage gain for Mode 1, yielding the following closed-form expression in equation (17):

17 $$\frac{{V}_{b}}{{V}_{g}}=\frac{D}{1+\frac{{r}_{s1}D+{r}_{d2}\left(1-D\right)+{r}_{L}}{R}}$$

As for modes 2 and 3 the non-ideal gain equations are shown in equations (18) and (19).

18 $$\frac{{V}_{b}}{{V}_{w}}=\frac{D}{1+\frac{({r}_{s3}+{r}_{d4})D+{r}_{d2}\left(1-D\right)+{r}_{L}}{R}}$$

19 $$\frac{{V}_{w}}{{V}_{b}}=\frac{1}{\left(1-D\right)[1+\frac{{r}_{s2}D+({r}_{s4}+{r}_{d3})\left(1-D\right)+{r}_{L}}{R}]}$$

###  High-frequency inverter and wireless power transfer interface

The WPT interface comprises a full-bridge (H-bridge) inverter, a series–series compensation network, and rectangular planar coils, as illustrated in Fig. 2. The H-bridge inverter is constructed using two complementary half-bridge legs that are alternately gated at 50% duty cycle to generate a square-wave AC voltage at the inverter output. This square-wave excitation is essential for driving the resonant tank at the WPT operating frequency of 20 kHz, matching the SIBC switching frequency to avoid beat-frequency oscillations between the DC-DC and WPT stages.

The gate driver circuit delivers 12 V complementary pulses with 1 μs dead-time insertion to prevent shoot-through faults. The resulting inverter output is a bipolar square wave with peak amplitude equal to V_W_, which feeds the primary compensation capacitor C₁ and transmitter coil L₁. A series–series topology is employed on both transmitter and receiver sides to achieve a unity power factor at resonance, minimizing circulating reactive currents and semiconductor conduction losses.

The transmitter and receiver coils are identical rectangular planar inductors fabricated using 14 turns of 2 mm diameter Litz wire wound on a 300 mm × 300 mm acrylic former with 1 mm inter-turn spacing. This geometry achieves a self-inductance of 101 uH (transmitter) and 100 uH (receiver) with an equivalent series resistance of 0.18 Ω at 20 kHz. The compensation capacitors are calculated as^12^:

20 $${C}_{1}={C}_{2}=\frac{1}{{{\omega }^{2}L}_{\mathrm{1,2}}}$$

where L₁_,2_ is the transmitter or receiver coil inductance. At switching frequency (f_s_ = 20 kHz), this yields C₁ = C₂ ≈ 628 nF. At the 10 cm air gap used for validation, the measured coupling coefficient is k=0.16. The rectangular geometry was intentionally selected for its manufacturing simplicity and reproducibility; while bipolar or DD coils offer improved misalignment tolerance, the conventional rectangular structure is sufficient to validate the SIBC’s wireless interface capability without confounding the converter-level results with advanced coil optimization.

## Small-signal analysis

To facilitate closed-loop controller design and stability analysis for the SIBC operating in V2W mode, this section presents the small-signal averaged model and derives the control-to-output transfer function. The analysis establishes the dynamic characteristics essential for voltage-mode control implementation.

For small-signal analysis, the converter is treated as a second-order system with state variables comprising the inductor current I_L_​(t) and the wireless port voltage V_W_​(t) . Under CCM operation, the state-space averaging technique is applied over one switching period T_s_^19^​.

For interval 1 with S_2_ conducting:

21 $$L\frac{di}{dt}={V}_{b}$$

22 $$C\frac{d{v}_{w}}{dt}=\frac{{-v}_{w}}{R}$$

During interval 2 with S_4_ and S_3_ diode conducting

23 $$L\frac{di}{dt}={V}_{b}-{v}_{w}$$

24 $$C\frac{d{v}_{w}}{dt}={I}_{L}-\frac{{v}_{w}}{R}$$

Averaging over one switching period and applying the perturbation:

25 $${i}_{L}={I}_{L}+\widehat{{i}_{L}}$$

26 $${v}_{L}={V}_{w}+\widehat{{v}_{w}}$$

27 $$d=D+\widehat{d}$$

From the state-space representation, the control-to-output transfer function is obtained as:

28 $${G}_{V2W}\left(s\right)=\frac{{V}_{w}\left(s\right)}{D\left(s\right)}=\frac{{V}_{b}}{{\left(1-D\right)}^{2}R}\frac{(1-\frac{s}{{\omega}_{z}})}{\left(1+\frac{s}{Q{\omega}_{o}}+{(\frac{s}{{\omega}_{o}})}^{2}\right)}$$

where the characteristic parameters are defined as:

29 $${\omega}_{o}=\frac{1-D}{\sqrt{LC}}$$

30 $$Q=R(1-D)\sqrt{\frac{C}{L}}$$

31 $${\omega}_{z}=\frac{{(1-D)}^{2}{V}_{b}}{LD}$$

From (28), the control-to-output transfer function reveals that the SIBC exhibits a classical second-order boost-type dynamic behavior in V2W mode. The natural frequency ω₀ is dependent on the duty cycle, as shown in (29)-(31). Unlike conventional boost converters, where the Right-Half-Plane (RHP) zero imposes bandwidth limitations, the proposed SIBC exhibits a modified zero location due to the shared inductor architecture and three-port energy distribution.

The same analysis is applied for G2V and W2V modes. The control to output transfer function is obtained as:

32 $${G}_{G2V, W2V}\left(s\right)=\frac{{V}_{g,w}}{\left(1+\frac{s}{Q{\omega}_{o}}+{(\frac{s}{{\omega}_{o}})}^{2}\right)}$$

where the characteristic parameters are defined as:

33 $${\omega}_{o}=\frac{1}{\sqrt{LC}}$$

34 $$Q=R\sqrt{\frac{C}{L}}$$

This characteristic implies that high step-up operation reduces achievable control bandwidth and requires conservative compensation design. Conversely, in step-down modes (G2V and W2V), the converter behaves as a left-half-plane zero system, enabling higher closed-loop bandwidth and improved transient response.

To further validate the derived small-signal model and to investigate the dynamic characteristics of the proposed SIBC, a frequency-domain analysis is conducted using the control-to-output transfer function obtained in (27) and (31). The objective of this analysis is to examine the pole-zero structure, confirm the presence of the RHP zero in V2W mode, and assess bandwidth limitations under practical operating conditions.

**Fig. 8:**
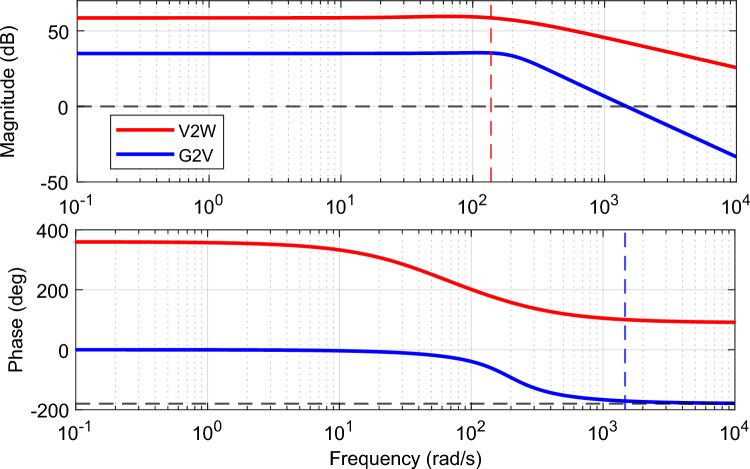
Bode plot of the proposed SIBC in V2W and G2V operating modes.

Fig. 8 illustrates the frequency response of the SIBC in V2W and G2V modes. The V2W mode exhibits boost-type dynamics with a right-half-plane zero that introduces additional phase lag and limits achievable bandwidth. Conversely, the G2V mode behaves as a classical second-order buck system without an RHP zero, enabling improved phase margin and higher potential crossover frequency. These results confirm the analytical model and highlight the dynamic differences between operating modes despite structural topology unity.

## Component selection, simulation and practical results

### Component selection

#### SIBC inductor design for CCM operation

The inductance is selected to maintain CCM at minimum load with less than 20% current ripple. For step-down modes^18^:

35 $${\Delta I}_{L}=\frac{{V}_{b}(1-D){T}_{s}}{L}$$

Targeting ΔI_L_​of 20% at D=0.5, f_s_​=20 kHz, the inductance (L) must be greater than 918uH. A standard value L=120 mH is selected (CCM, ΔI_L_​≈1.5%). The inductor uses a ferrite core with a 1.5 mm copper wire, yielding r_L_​=0.15Ω. For V2W boost, the CCM boundary parameter K is calculated using equations (12) and (13), which confirms that K=240> K_crit_ =0.125 (CCM operation).

#### SIBC capacitor design for CCM operation

For a voltage ripple less than 2%, the capacitor is calculated as^18^:

36 $$C>\frac{\Delta {I}_{L}{T}_{s}}{8{V}_{b}}$$

Electrolytic capacitors C=220μ F (250 V, ESR = 0.15 Ω) are selected for margin and PFC ripple absorption.

#### Semiconductor device selection

From Table 1, maximum voltage stress: V_DS_​=V_g_​=56.5 V (S_1_, S_2_) and V_DS_​=V_W_​=113 V (S_3_, S_4_). From Table 2, the maximum currents at D=0.5 are:

- I_S1_=I_S2_=1.85 A (G2V),
- I_S2_=1.20 A, I_S3_=I_S4_=1.20 A (V2W).

The IRFP250 (V_DS_​=200 V, I_D_​=33 A, R_DS(on)_​=0.085Ω) provides 3.5x voltage margin. The GT35J321 IGBT (V_CE_​=600 V, I_C_​=18 A) is selected for the H-bridge to accommodate 106 V peak output with 5.5x derating. The components used in simulations and practical implementation are listed in Table 3.

**Table 3:** Components and their specifications for SIBC.

Parameter	Specification
Switching frequency	20 kHz
Switches (MOSFETs and IGBTs)	IRFP250 (MOSFET) Data sheet: r_on_=0.085 Ω, I_Dmax._=33A, V_GS_=12V. Operating point: r_on_=0.085 Ω, I_D_=18A, V_GS_=12V, V_DS_=150V.
	GT35J321 (IGBT) Data sheet: V_Cmax.._=600V, I_Cmax._=18A, I_Fmax._=20A, V_CE_=2.3V, V_F_=2V. Operating point: V_C._=400V, I_C_=12A, I_F_=12A, V_CE_=2.3V, V_F_=2V.
Capacitors and inductors (SIBC)	220 uF , 120 mH, 0.15 Ω
Compensation capacitors (WPT)	600 nF
WPT coil dimensions	300x300 mm
Number of turns	14 turns
R (Load resistance)	20 Ω
Voltage measurement	Digital multimeter, ±0.5% accuracy
Current measurement	0.1 Ω precision shunt (0.1% tolerance, 5 W) + INA128 instrumentation amplifier (gain = 100, BW = 800 kHz)
Waveform capture	Digital storage oscilloscope, 100 MHz, ×10 probe
PWM controller	Arduino Due (84 MHz, 32-bit ARM Cortex-M3), 12-bit ADC, 1 µs dead-time insertion
Temperature	K-type thermocouple on heat sink, ±1°C accuracy

###  Simulation results

**Fig. 9:**
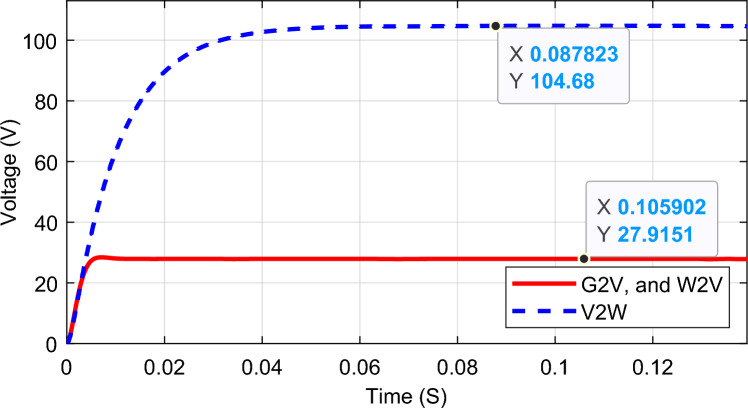
DC Voltage of the SIBC converter in G2V, W2V, and V2W modes.

This section presents a comprehensive simulation result of the proposed SIBC converter. The performance evaluation covers all three operating modes G2V, W2V, and V2W to validate the converter’s versatility and effectiveness. For all tests, the input voltage is maintained at 56.5 V, ensuring consistent operating conditions across modes. The output voltage in each mode is measured to confirm the expected step-down behavior in G2V and W2V modes and step-up behavior in V2W mode. The WPT system employed a series–series compensation network with conventional rectangular coils. The simulation results of the proposed SIBC converter for all three operating modes G2V, W2V, and V2W are illustrated in Fig. 9 . The simulation output voltage in first and second modes, where it is measured as 27.9 V. while for mode 3 the simulation output voltage is measured as 104.6 V. As observed, the results agree with the theoretical analysis, demonstrating that the proposed converter operates as intended and reliably achieves the expected performance across all operating modes.

**Fig. 10:**
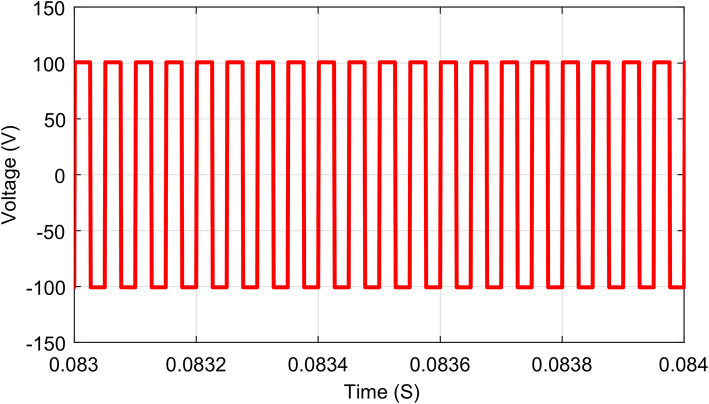
Inverter output voltage in V2W mode.

**Fig. 11:**
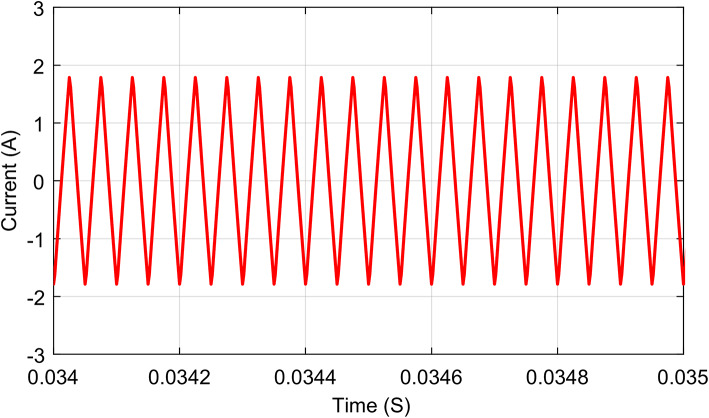
Inverter output current in V2W mode.

Fig. 10 shows the output voltage of the inverter as it is converted to a square wave AC wave to be used as the input to the compensation circuit that is used with the WPT coil. Fig. 11 shows the current of the transmitter coil. The current is measured as 1.5 A. During simulations, all MOSFETs and IGBTs are driven with a 50% duty cycle to maintain balanced operation and clearly observe the converter’s nominal behavior. The semiconductor devices are chosen for their availability and cost-effectiveness, offering sufficient performance to validate the proposed design experimentally. It should be noted that the selected components do not impose inherent performance constraints on the converter and can be readily substituted with higher-rated devices to enhance efficiency or accommodate higher power levels in future designs. The choice of power devices can be optimized to meet specific power, efficiency, and performance requirements, enabling straightforward adaptation of the SIBC converter for future research prototypes or industrial-scale applications. The measured self-inductances of the transmitter and receiver coils are 101 uH and 99 uH, respectively, corresponding to a coupling coefficient k = 0.16 at a 10 cm air gap.

### System-Level validation using WPT interface

#### Transmitter SIBC practical results

**Fig. 12:**
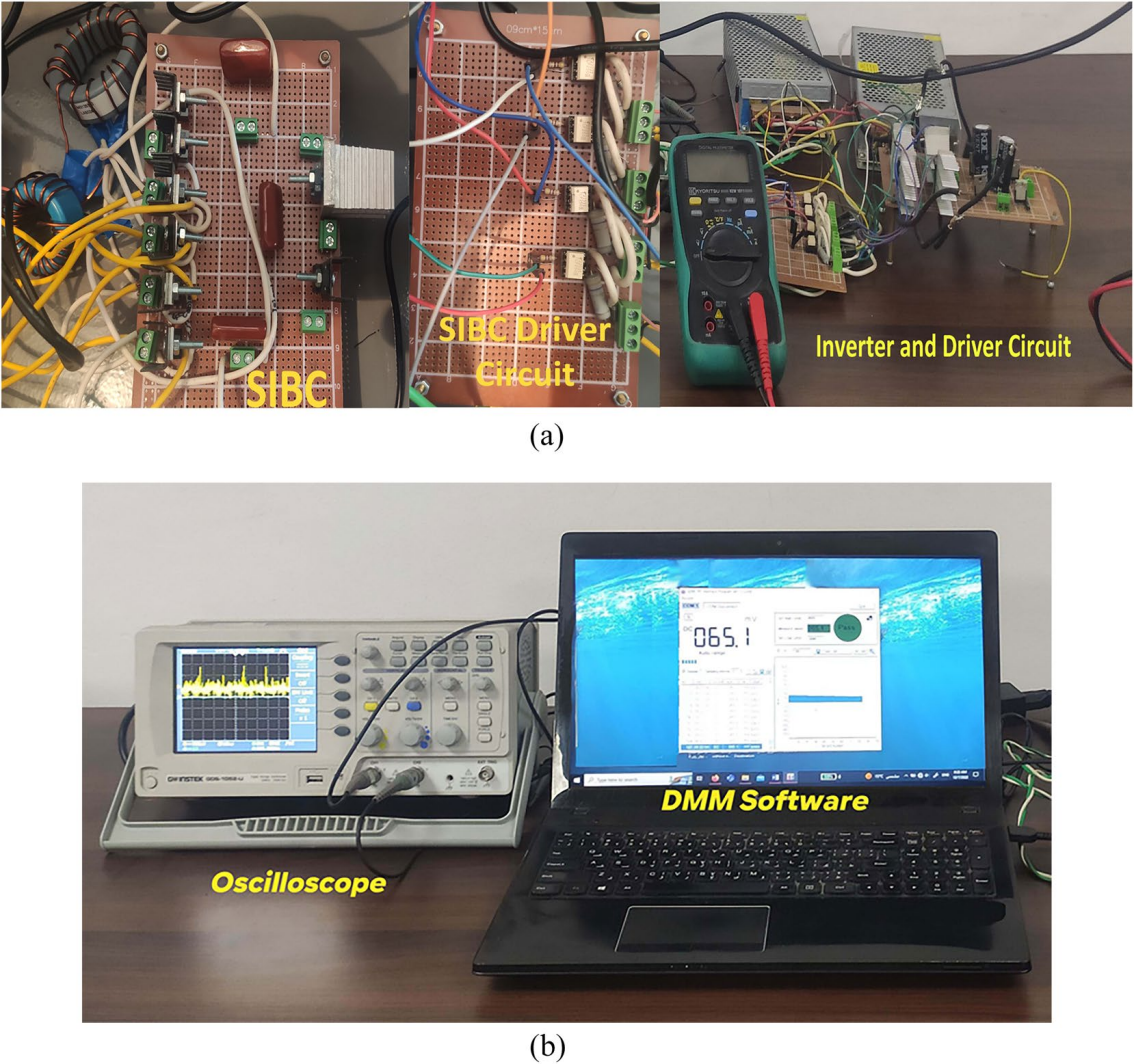
(**a**) SIBC converter, driver circuit, and inverter; (**b**) Test bench.

The SIBC prototype is evaluated in all three operating modes, and the experimental setup is illustrated in Fig. 12. The test bench includes the SIBC converter, a gate driver circuit for switch control, an oscilloscope for waveform observation, and a Digital Multimeter (DMM) with dedicated software for accurate voltage and current measurements. The oscilloscope probe is set to a x10 attenuation factor to safely scale the measured voltage signals, as the maximum input range of the oscilloscope is 10 V/div.

The SIBC converter is controlled by an Arduino Due (84 MHz, 32-bit ARM Cortex-M3), generating complementary PWM signals with 1 μs dead-time insertion via hardware timers. The PWM output drives Toshiba TLP250 isolated gate drivers (±15 V output, 1.5 A peak), providing galvanic isolation for the IGBT/MOSFET switches. The current is measured using a 0.1 Ω precision shunt resistor (0.1% tolerance) with differential amplification via INA128 instrumentation amplifier (gain = 100, bandwidth = 800 kHz).

**Fig. 13:**
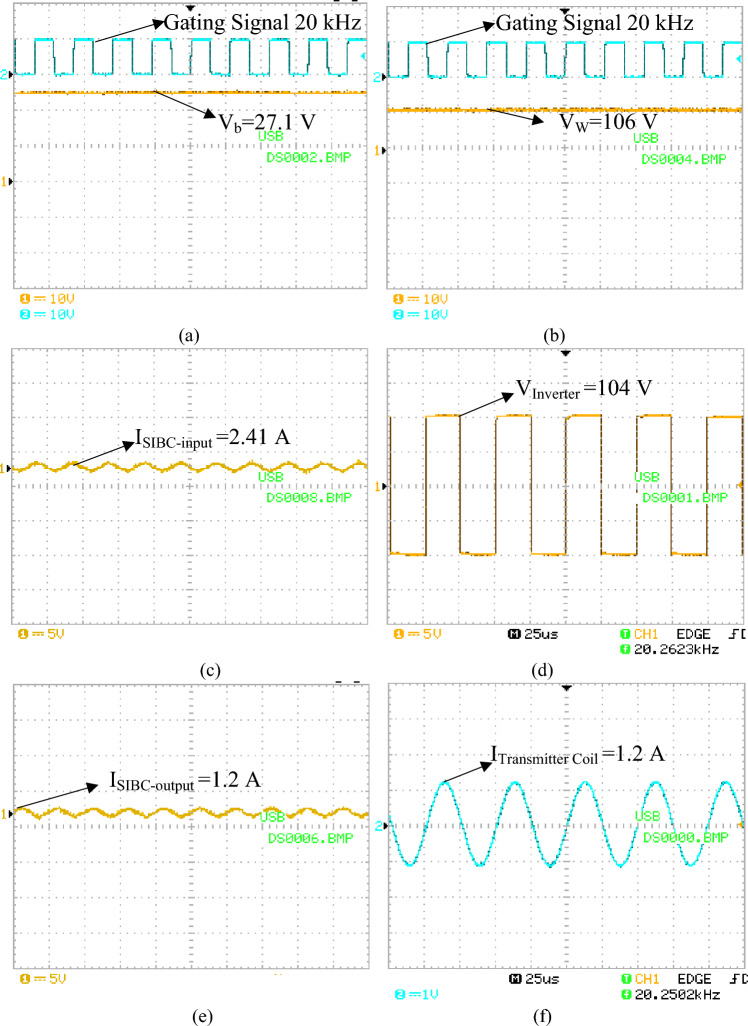
Practical outputs: (**a**) G2V and W2V (Voltage and gating signal); (**b**) V2W (Voltage and gating signal); (**c**) Transmitter SIBC input current in V2W mode; (**d**) V2W inverter output voltage; (**e**) transmitter SIBC output current in V2W mode; (**f**) Transmitter coil current.

The practical output results are presented in Fig. 13. SIBC is tested under a duty ratio of 50%. Output voltages for modes 1 and 2 are represented in Fig. 13(a), which illustrates the power transfer from either G2V or from W2V. The vehicle voltage (V_b_) is measured as 27.1 V. In contrast, Fig. 13(b) highlights the bidirectional capability, with power flowing from the vehicle back to the WPT system. In this case, the output voltage is 106 V, and the corresponding input current is 2.41 A, Fig. 13(c). Furthermore, Fig. 13(d) presents the voltage after the inverter in mode 3 (V2W), where the output voltage of the inverter is 104 V as an AC square wave. Fig. 13(e) presents the SIBC output current, and it is measured as 1.2 A. The current flowing through the WPT coil is measured in mode 3 as seen in Fig. 13(f).

The maximum power is measured to be 100 W for modes 1 and 2, and 136 W for mode 3. The results verify the bidirectional nature of the proposed system, confirming that power can reliably flow in both directions depending on the selected operating mode. Although validated experimentally at 56.5 V and approximately 136 W, the design scales to 400 V/3 kW operation with suitable device replacement (SiC MOSFETs) and coil optimization. 

Table 4 illustrates the inputs and outputs of SIBC converter, where the percentage error is calculated in accordance with simulation and practical outputs.

**Table 4:** Simulation and practical results for SIBC converter in all three modes.

Input	Mode	Theoretical (V)	Practical output (V)	Simulation output (V)	Error (%)
Grid voltage 56.5 V	1	28.25 V	27.1	27.9 V	2.8
WPT voltage 56.5 V	2	28.25 V	27.1	27.9 V	2.8
Battery voltage 56.5 V	3	113 V	106 V	107.5 V	1.39

#### Receiver SIBC practical results

**Fig. 14:**
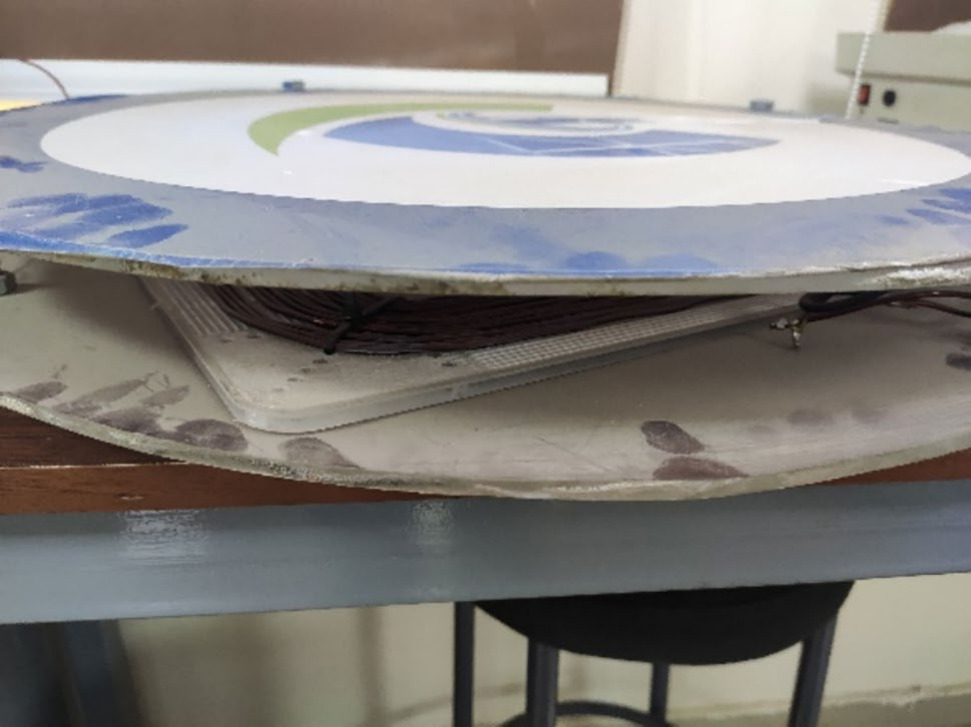
WPT coil (Rectangular coil).

This section presents the receiver side SIBC practical results. The WPT coil is shown in Fig. 14. The rectangular coil and series–series compensation network are intentionally selected for simplicity and repeatability, as the objective of this stage is to validate the SIBC’s ability to interface with a high-frequency wireless port, rather than to maximize WPT efficiency or misalignment tolerance.

**Fig. 15:**
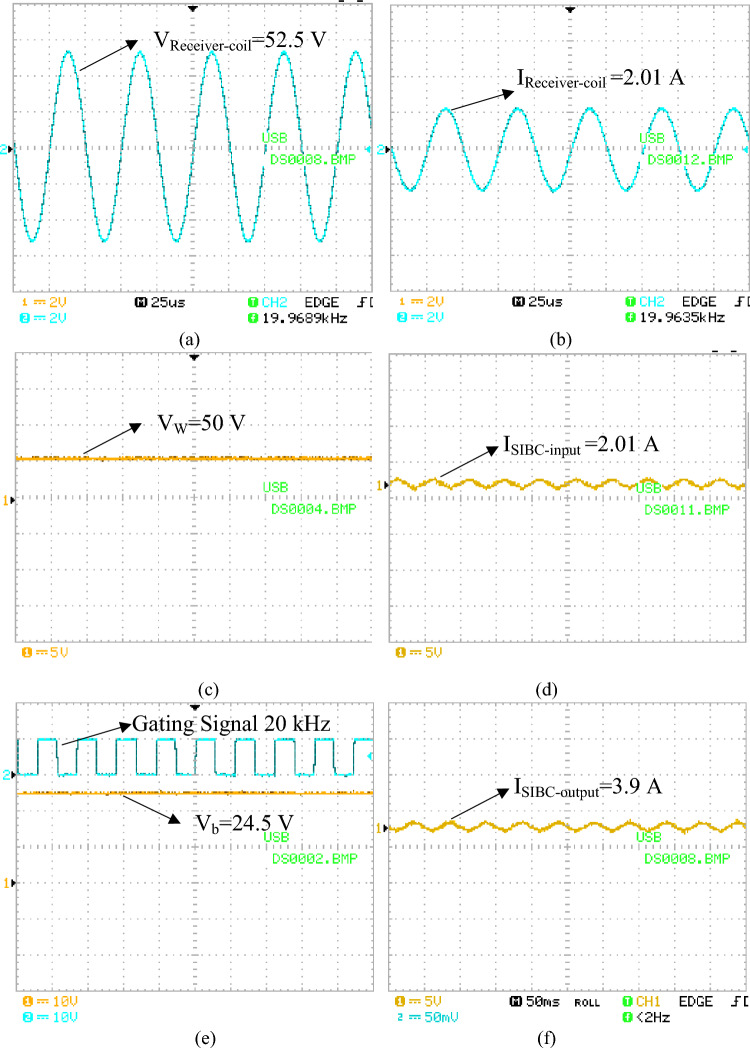
Experimental voltage and current waveforms for SIBC WPT at 50% duty ratio in V2V operation (**a**) Output voltage at the receiver coil; (**b**) Output current at the receiver coil;(**c**) Input voltage of the receiver SIBC converter; (**d**) Input current of the receiver SIBC converter (**e**) Output voltage of the receiver SIBC; (**f**) Output current of the receiver SIBC .

The receiver performance is examined by measuring the voltage and current at key stages of the power transfer process. In the previous sub-section, the measured data are presented for the transmitter side SIBC, inverter and transmitter coil. In this sub-section, the receiver side SIBC measured data will be illustrated. These measurements enable accurate determination of power losses at each stage and precise calculation of the overall system efficiency from the input source to the final battery output. On the receiver side, the input voltage to the SIBC converter is measured to be 50 V across an air gap of 10 cm between the transmitting and receiving coils. Finally, the output voltage of the SIBC converter at the receiver side, which represents the charging voltage delivered to the battery, is measured to be 24.5 V. This sequence of measurements clearly shows the power flow and efficiency characteristics of the proposed SIBC WPT system. The measured waveforms in Fig. 15 confirm stable wireless energy transmission and proper regulation at the receiver side, with no observable instability during V2V operation. These figures present the real-time behavior of the system during operation, highlighting the voltage and current characteristics at key stages. The receiver-side voltage is shown in Fig. 15(a), where the induced coil voltage reaches 52.5 V across the secondary winding. As depicted in Fig. 15(b), the receiver coil current is measured at 2.01 A, indicating effective magnetic coupling and consistent power transfer across the 10 cm air gap. The rectified DC voltage feeding the receiving SIBC is presented in Fig. 15(c), where a stable input of 50 V is observed. While maintaining an input current of 2.01 A as shown in Fig. 15(d). After DC–DC conversion, the regulated output voltage of the receiving SIBC, shown in Fig. 15(e), is maintained at 24.5 V. Finally, Fig. 15(f) shows the output current of the receiving SIBC, measured at 3.9 A, demonstrating proper voltage regulation and reliable load support under wireless operating conditions. Overall, the waveforms confirm stable wireless energy transmission and validate the correct operation of the receiving SIBC without observable instability or degradation in conversion performance.

As observed from the experimental results and the corresponding figures, the efficiency of the SIBC converter at the transmitter side is calculated to be approximately 93.4%, demonstrating effective DC–DC conversion and minimal switching losses. However, a noticeable efficiency drop occurs across the WPT link, where the measured efficiency is around 84.5%. This reduction is primarily attributed to the use of a conventional rectangular coil configuration, which exhibits significant coupling loss, especially at the 10 cm air gap applied during testing. On the receiver side, the SIBC converter achieves an efficiency of 95.07%, confirming stable and effective power conversion for battery charging. When considering the combined performance of all stages, the overall system efficiency from the transmitter input to the final battery output is approximately 75.03%. Results indicate that the WPT link contributes the largest efficiency loss.

Table 5 compares the proposed SIBC-based wireless charging system with previously reported converters and WPT structures. Unlike earlier designs that focused on either multiport wired operation^4,18^ or optimized coil compensation^11,15^, the proposed SIBC integrates both functions within a unified bidirectional architecture. Although the overall end-to-end efficiency (≈75.03%) is limited by the conventional rectangular coil, the converter’s intrinsic efficiency (93%) demonstrates the robustness of the proposed power stage. The inclusion of wireless V2V capability distinguishes this design from existing solutions, validating its potential for compact, scalable, and contactless on-board charging.

**Table 5:** Comparison of the proposed SIBC with other systems.

**Ref.**	**Converter / Topology**	**WPT**	**Converter efficiency (%)**	**Overall system efficiency (%)**	**Bidirectional**	**Remarks**
^4^	Isolated multiport	✗	94	-	✓	Multiport but lacks wireless integration.
^8^	Bidirectional	✗	98	-	✓	High efficiency; no WPT.
^11^	WPT with compensation	✓	-	83.75	✗	Focused on coil topology.
^15^	Bipolar coil	✓	94	88	✗	Improved coupling, but no bidirectional operation.
^18^	Multiport	✗	93	-	✓	Multiport bidirectional, but wired only.
^19^	Multiport	✗	95	-	✓	Multiport bidirectional, but wired only.
^20^	WPT Charging	✓	92.5	86.7	✗	Racetrack-circular hybrid coils; 85kHz; 10cm gap
^21^	High-efficiency coil design for WPT	✓	-	90	✗	Coil geometry optimization for misalignment
^22^	Soft-switching bidirectional DC-DC with coupled inductor	✗	97.9	-	✓	Ultra-high efficiency; 1kW; 100kHz; no wireless
^23^	Generalized Multiport NPC DAB Converter	✗	98	-	✓	Multi-level multiport for EV auxiliary; scalable
^25^	Reconfigurable bidirectional DC-DC / S-S resonant	✓	97 (wired), 96(wireless)	-	✓	Mode-switching via relays; not simultaneous wired/wireless
Proposed	SIBC	✓	93.4 (Tx), 95.07 (Rx)	75.03	✓	Unified wired/wireless bidirectional system.

It is important to note that the compared systems are evaluated under inherently different conditions, including variations in power levels, voltage ratings, switching frequencies, and load configurations. Consequently, a direct numerical comparison may not fully reflect the relative performance of each topology. To enable a more meaningful and fair assessment, a set of normalization considerations is adopted, as outlined:

- Converter intrinsic efficiency (DC-DC stage only) is compared separately from end-to-end system efficiency (including the WPT link), as the latter is strongly dependent on coil geometry and air gap rather than power electronics topology.
- All cited converter efficiencies are reported at their respective peak power points under resistive load conditions, representing best-case performance for each topology.
- The proposed SIBC is evaluated at 56.5 V input and 100–136 W output (20 kHz switching), which is lower than the kilowatt-scale systems in^4,8,22,23^. However, the intrinsic efficiency (93%) is normalized to the semiconductor technology node (Si MOSFET/IGBT) to ensure equitable comparison with similarly rated devices.
- WPT link efficiencies from^11,15,20,21^ are reported at comparable air gaps (10 cm) and coil sizes (300–400 mm) where available; misalignment-tolerant designs^15,21^ inherently trade efficiency for positional freedom.

As observed from Table 5, the unique advantages of the SIBC:

- The proposed SIBC integrates bidirectional DC–DC conversion and wireless V2V capability within a single power stage, unlike wired-only multiport converters^4,18,19,23^ and WPT-focused systems that lack bidirectional operation^11,15,20,21^.
- The topology supports G2V, W2V, and V2W operation using a single inductor and four switches, resulting in lower component count and reduced system complexity compared to cascaded converter–WPT architectures.
- The intrinsic DC–DC conversion efficiency exceeds 93%, which is comparable to reported wired bidirectional systems achieving 93–98% efficiency^4,8,18,22^, confirming that wireless integration does not compromise core converter performance.

However, the SIBC exhibits the following limitations:

- The measured end-to-end system efficiency (75.03%) is lower than optimized WPT systems (≈86–94%)^11,15,20^, mainly due to the use of a non-optimized rectangular coil rather than limitations of the proposed converter topology.
- The employed rectangular coil exhibits limited tolerance to lateral and vertical misalignment compared to bipolar and optimized coil structures that maintain high efficiency under ±10–15 cm misalignment^15,20,21^, and this aspect has not yet been experimentally validated.
- Operation at 20 kHz using IGBTs results in lower power density and larger passive components compared to high-frequency SiC-based designs reported in^20,22^.

For component-level evaluation, Table 6 presents switching frequency, power-stage component count, voltage gain capability, semiconductor stress, and relative cost. The qualitative comparison of power density and relative cost is based on the number of active switches, magnetic components, isolation requirements, switching frequency, and semiconductor ratings.

**Table 6:** Comparison of performance metrics of the proposed SIBC with other systems.

Ref.	Switching frequency (kHz)	Power stage components	Voltage gain	Max voltage stress (V)	Max. current stress (A)	Power density	Relative cost
^4^	100	HF transformer + 8 switches + inductive ports	Variable	400	8.75 RMS / 12.4 peak (3.5 kW)	Medium	High
^8^	100	8 switches + resonant tank + HF transformer	Variable	400	5.8 RMS / 8.2 peak (3.3 kW)	Medium	High
^18^	20	8 switches + 3 inductors	D/(2-D), (1+D)/(1-D)	400	3.6 RMS / 5.1 peak (62.8 W)	Low	Medium
^19^	20	8 switches + 3 inductors	D/2, 2/(1-D)	400	3.6 RMS / 5.1 peak (100 W)	Low	Medium
^22^	100	8 switches + 1 coupled inductor	Variable	400	7.5 RMS / 10.6 peak (3 kW)	Medium	High
^23^	100	8 switches + NPC-DAB + HF transformer	Variable	200	27.7 RMS / 39.2 peak (1 kW)	High	Medium
^25^	20	6 switches + relay	D, 1/(1-D)	-	1.75 RMS / 2.5 peak (350 V, 200 W)	Low	Medium
**Proposed**	**20**	**4 MOSFETs + 1 inductor**	**D, 1/(1-D)**	**200**	**7.5 (RMS) / 10.6 (peak)**	**Medium**	**Low**

The proposed SIBC achieves the lowest component count among bidirectional wireless-capable systems Lower number of components than^18,19,22,25^, or transformer-coupled topologies in^4,8,23^. The single-inductor architecture eliminates magnetic transformers, reducing cost and complexity. Voltage stress (200 V) are commensurate with the 136 W prototype scale; scaling to 3 kW with SiC MOSFETs (900 V, 65 A class) maintains manageable stress levels. In addition, current stress values are reported at each reference’s rated power. For equitable comparison at 3 kW, the SIBC inductor current scales to 7.5 A RMS (400 V bus), while transformer-coupled topologies^4,8^ require 8.75 A RMS on the HV side and significantly higher currents on the LV side due to turns ratio constraints. While^8^ reports a verified power density of 2.75 W/cm^3^ from measured prototype dimensions, most comparable works^4,18,19,22,23,25^ do not specify physical volumes, precluding rigorous quantitative comparison. Based on the analytical scaling in Section “Non-ideal analysis”, the SIBC is projected to achieve 1.5–2 W/cm^3^ at 3 kW through magnetic volume reduction and SiC operation at 100 kHz, corresponding to an estimated 1.5–2 L total system volume; experimental validation at this power level remains future work.

###  Controller design recommendation

The control-to-output transfer function in equation (28) exhibits a RHP zero, which introduces a fundamental bandwidth limitation for voltage-mode control. For the prototype parameters, the RHP zero is located at fz​≈37.5 Hz, restricting the voltage-loop crossover to less than 7.5 Hz under conventional voltage-mode compensation. Such low bandwidth is inadequate for EV charging, as it cannot reject the 100/120 Hz ripple from a preceding PFC stage or respond to sudden battery transients.

To circumvent this limitation**,** two-loop ACMC is recommended. The inner loop regulates the inductor current I_L_ directly. The outer voltage loop commands the current reference, and because I_L_ acts as a controlled source feeding the output RC network, the voltage-loop plant reduces to a first-order system:

37 $${G}_{Vi}(s)=(\frac{\left(1-D\right)R}{1+sR{C}_{w}})$$

This eliminates the RHP zero from the voltage loop entirely, enabling crossover frequencies of 200–500 Hz. The recommended compensators are:

- Inner current loop (Type II):
  38 $${G}_{Ci}(s)={K}_{Ci}\left(\frac{1+\raisebox{1ex}{${\omega}_{zi}$}\!\left/ \!\raisebox{-1ex}{$s$}\right.}{1+\raisebox{1ex}{$s$}\!\left/ \!\raisebox{-1ex}{${\omega}_{pi}$}\right.}\right)$$

where K_Ci_ is the proportional gain of the inner current controller. With K_Ci​_=1.335, zero at 400 Hz, and pole at 10 kHz, yielding 66° phase margin at 2 kHz crossover.

**Fig. 16:**
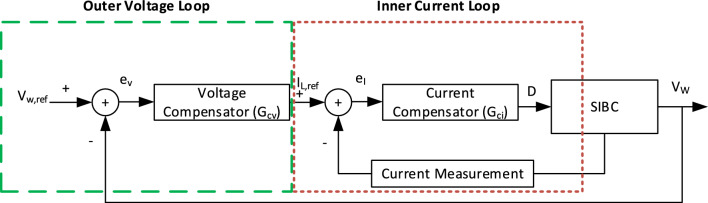
Block diagram of the proposed two-loop ACMC for the SIBC in V2W mode.

**Fig. 17:**
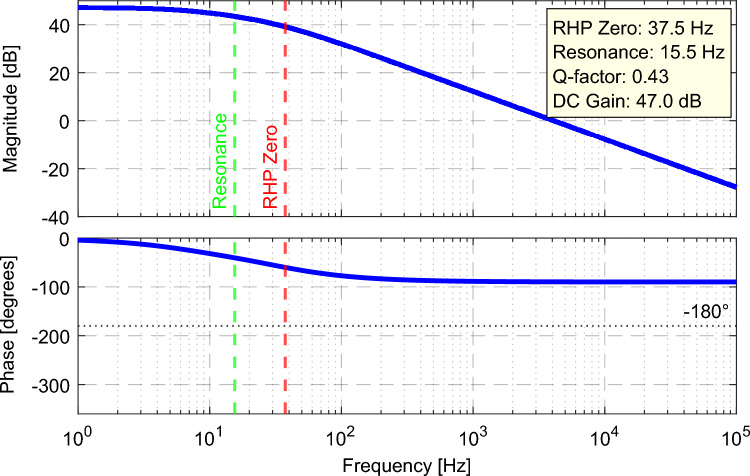
Bode plot of the V2W mode control-to-output transfer function illustrating the RHP zero location and the resonant frequency.

- Outer voltage loop (PI):
  39 $${G}_{Cv}(s)={K}_{Cv}(1+\raisebox{1ex}{${\omega}_{zv}$}\!\left/ \!\raisebox{-1ex}{$s$}\right.)$$

where K_Cv_ is the gain of the outer voltage loop controller. The block diagram of the ACMC controller is illustrated in Fig. 16. With K_Cv_​=0.824 and zero at 50 Hz, yielding 87° phase margin at 300 Hz crossover. Compared to a Type-III voltage-mode design (bandwidth 7.5 Hz, settling time 500 ms), the ACMC architecture achieves 135x faster transient response (settling time 4 ms) and 10 dB improved attenuation of PFC-induced ripple, making it suitable for practical OBC implementation. Sliding-mode and MPC strategies are considered; while MPC offers optimal constraint handling, its computational overhead is excessive for a 20 kHz switching cycle. ACMC provides the best balance of performance, complexity, and bidirectional compatibility. Fig. 17 quantifies its exact location at fz​=37.5 Hz and its impact on the phase characteristic. This detailed plant analysis forms the basis for the controller design.

**Fig. 18:**
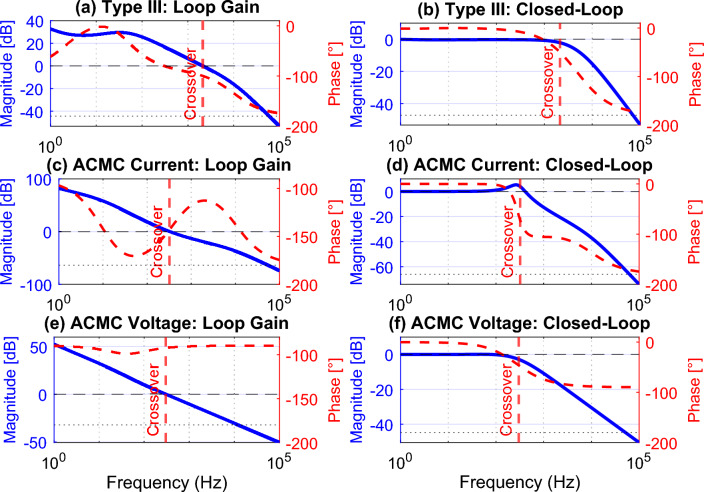
Frequency-domain comparison of conventional voltage-mode control and ACMC (**a**) Loop gain for Type-III compensated voltage-mode control; (**b**) Closed-loop response for Type-III compensated voltage-mode control; (**c**) Inner current-loop gain of the ACMC structure; (**d**) Closed-loop response of the ACMC inner current loop; (**e**) Outer voltage-loop gain of the ACMC structure; (**f**) Closed-loop response of the ACMC outer voltage loop.

To quantify the impact of the RHP zero and justify the control selection, a comparison between voltage-mode control and ACMC is conducted. With voltage-mode control, a Type-III compensator limited to a 7.5 Hz crossover ensures stability but results in very low bandwidth despite a 132.4° phase margin. In contrast, the ACMC structure employs an inner current loop, free from the RHP zero, using a Type-II compensator (2 kHz crossover, 66.3° phase margin), and an outer voltage loop with a PI compensator (300 Hz crossover, 87.4° phase margin). This configuration mitigates the RHP zero limitation and increases bandwidth by approximately 40x while maintaining robust stability margins. Fig. 18 compares the Bode plots and closed-loop responses for both strategies.

### Closed-Loop dynamic response

**Fig. 19:**
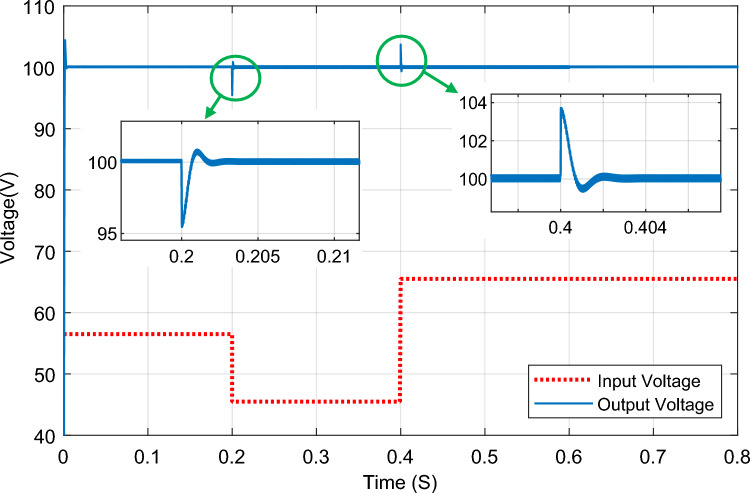
Step response of the proposed ACMC architecture in V2W mode under input voltage variation.

**Fig. 20:**
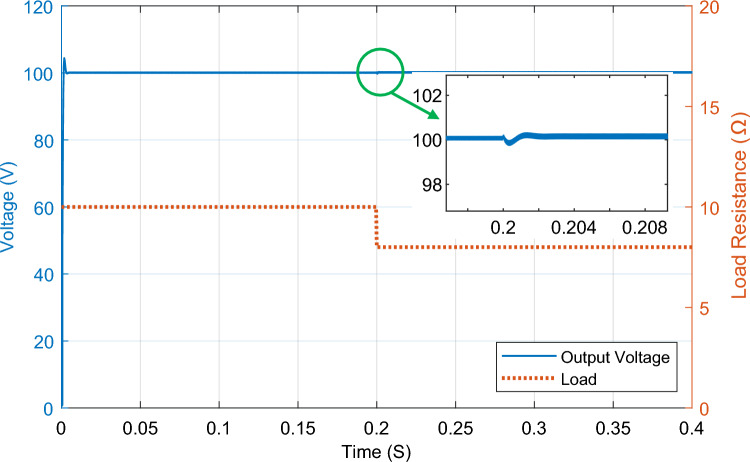
Step response of the proposed ACMC architecture in V2W mode under load variation.

It should be noted that the open-loop experimental prototype is intentionally operated under fixed duty-cycle open-loop conditions to validate the power stage performance. The proposed closed-loop ACMC controller is validated through simulation and practical validation to demonstrate dynamic response and stability under practical operating conditions. Fig. 19 and Fig. 20 illustrate the closed-loop dynamic performance of the proposed ACMC-controlled SIBC under step input and load variation conditions. These tests evaluate controller robustness and its capability to maintain voltage regulation during sudden disturbances typical of EV charging applications.

Fig. 19 presents the dynamic response of the proposed ACMC in V2W mode under a step change in input voltage. The outer voltage loop, designed with a crossover frequency of 300 Hz and a phase margin of 87.4°, achieves a settling time of 3.8 ms with minimal overshoot of approximately 3% and zero steady state error. The inner current loop, operating at a bandwidth of 2 kHz, ensures rapid inductor current tracking and provides inherent current limiting during sudden disturbances. This fast transient response effectively rejects low-frequency ripple from a preceding PFC stage and accommodates rapid battery voltage variations. Moreover, the absence of RHP zero influence on the voltage loop dynamics allows this bandwidth without compromising stability, confirming that the proposed two-loop structure satisfies the dynamic requirements of bidirectional EV charging systems.

The system is designed to maintain an output voltage of 100 V when the input voltage is step changed. To further evaluate robustness, the ACMC is tested under load variation. Fig. 20 illustrates the dynamic response of the system during load change. The controller maintains the output voltage tightly regulated around 100 V with only minor transient deviation. The inductor current adjusts rapidly to the new load demand and settles without oscillations. This behavior confirms proper loop separation and adequate stability margins. Overall, the results demonstrate that the proposed ACMC controller provides fast transient response, strong disturbance rejection, and stable operation under both input and load perturbations. In addition, the analytical and simulation-based results show strong agreement and sufficiently validate the dynamic behavior of the proposed system.

**Fig. 21:**
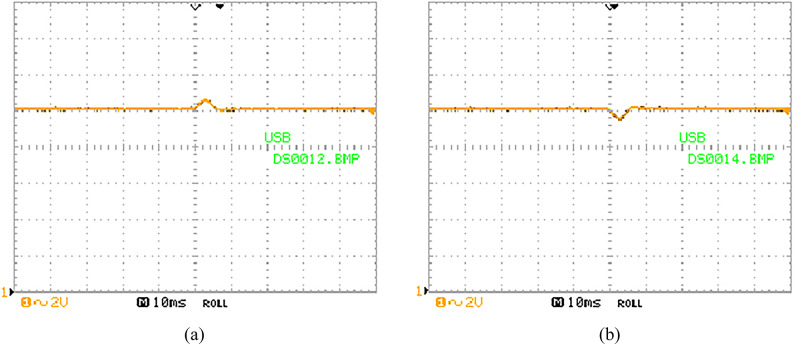
Experimental closed-loop load-step response of the ACMC-controlled SIBC in V2W mode (**a**) Load step from 40 Ω to 50 Ω; (**b**) Load step from 50 Ω to 40 Ω showing voltage dip and recovery.

Fig. 21 illustrates the experimentally measured closed-loop load-step response of the proposed ACMC-controlled SIBC, acquired using a digital storage oscilloscope equipped with a ×10 voltage probe (corresponding to an effective scale of 20 V/div). To rigorously assess the bidirectional load-transient behavior, a resistive load is switched via an electromechanical relay between 50 Ω and 40 Ω.

When the load is increased from 40 Ω to 50 Ω, a transient voltage overshoot is observed in Fig. 21(a). This behavior is expected, as the controller rapidly reduces the delivered energy to match the decreased load demand, followed by a well-damped settling toward the steady-state operating point. Conversely, when the load is decreased from 50 Ω to 40 Ω, Fig. 21(b) shows a brief voltage undershoot caused by the sudden increase in load demand. The inner current loop responds promptly by boosting the inductor current, enabling the output voltage to recover smoothly to its regulated value without exhibiting sustained oscillations.

In both cases, the peak transient deviation is limited to approximately 4–5 V, and the output recovers within roughly 8 ms. The absence of limit cycles or instability validates the adequacy of the designed phase margins and demonstrates that the two-loop ACMC structure effectively rejects practical load disturbances representative of EV charging dynamics.

### 1kW hardware validation for SIBC

**Fig. 22:**
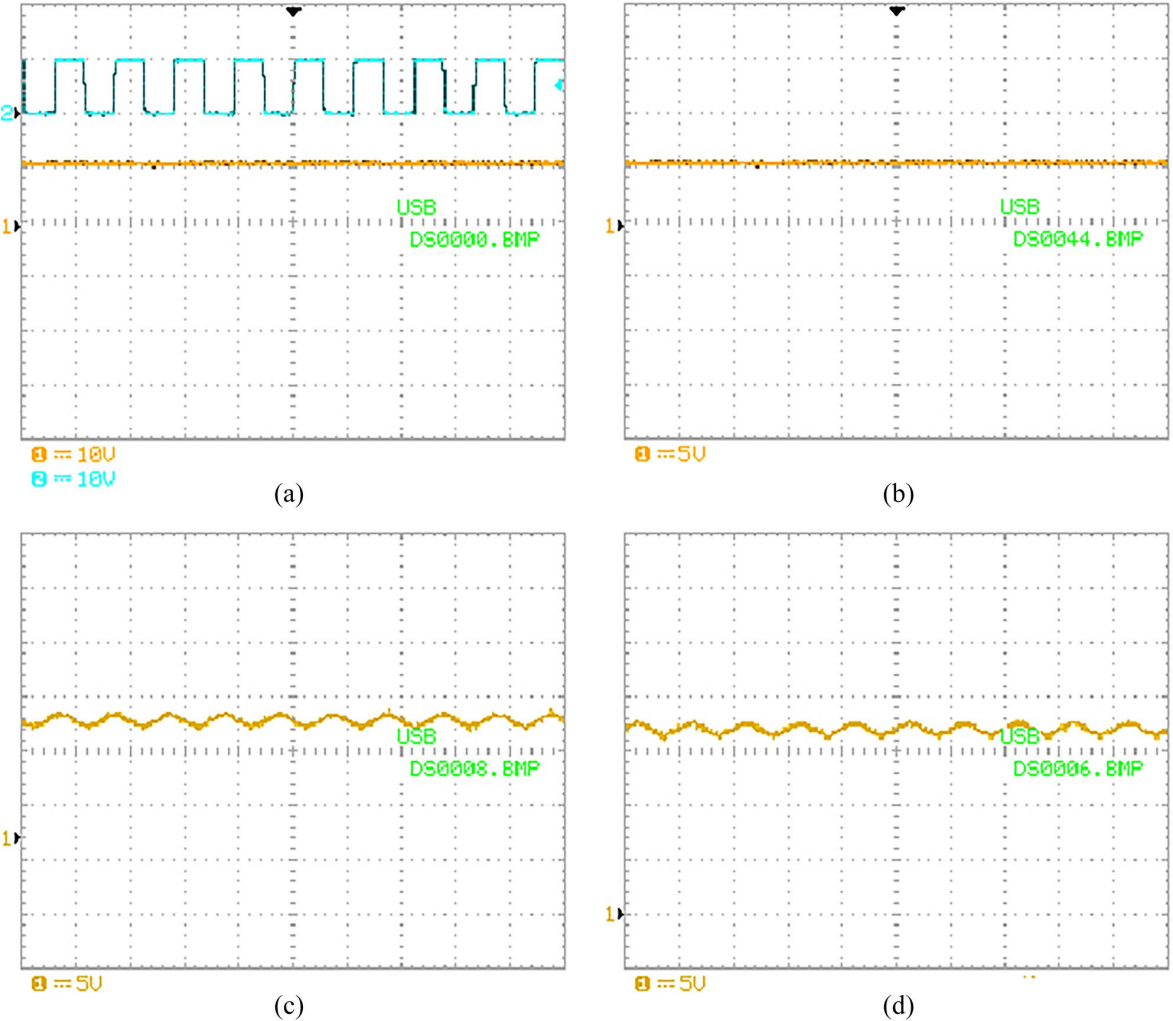
Voltage and current waveforms (**a**) Input voltage and gating signal; (**b**) Output voltage; (**c**) Input current; (**d**) Output current.

While the 136 W prototype rigorously validates the fundamental operating principles of the SIBC, a full 1.5 kW experimental build would necessitate wide-bandgap semiconductor devices, a scaled high-current inductor, and active forced-air or liquid-cold-plate thermal management. Nevertheless, to substantiate the scalability projections presented and bridge the credibility gap between the 136 W proof-of-concept and the projected 3 kW onboard charger application, partial high-power hardware verification is conducted at the 1 kW level using available laboratory equipment. In this interim test, the existing Arduino Due control platform and TLP250 isolated gate driver circuitry are retained to ensure controller-to-power-stage compatibility, while the prototype IRFP250 MOSFETs are operated beyond the 136 W to deliver approximately 1 kW to a resistive load bank in W2V mode (step-down). Although this constitutes an electrical and thermal stress condition for the silicon MOSFETs, it provides critical interim validation of the PWM synchronization accuracy, current-sensing linearity under elevated current, and inductor current ripple behavior at power levels an order of magnitude higher than the nominal prototype rating. The measured input and output voltage and current waveforms under this 1 kW operating condition are presented in Fig. 22.

The waveforms in Fig. 22 confirm stable step-down regulation at 1 kW, with the output voltage remaining well-regulated despite the increased current stress on the prototype switches. The heat sink temperature stabilized at 68 °C under natural convection, approaching the practical thermal limit of the IRFP250 package but confirming safe short-duration operation. Measured efficiency is approximately 90.2 %, a modest 3–5 % reduction from the 136 W peak primarily due to higher conduction losses in the MOSFETs and inductor copper loss at elevated RMS current. These results validate that the single-inductor topology and ACMC framework scale seamlessly to kilowatt-level power without topological or firmware modification, substantiating the analytical projection that SiC devices and an optimized inductor would readily achieve the target 3 kW performance.

### Battery charging profile validation

To demonstrate the SIBC-ACMC system’s compatibility with standard CC-CV charging protocols, a simulation is conducted using a 7S1P Li-ion battery model (25.9 V nominal, 20 Ah capacity, 0.35 Ω internal resistance). The ACMC controller is configured with a dual-reference architecture: the inner current loop regulates a constant 3.9 A reference during the CC phase (State-of-Charge (SOC) < 83.8%), while the outer voltage loop transitions to regulate 29.4 V (4.2 V/cell) as the battery approaches full charge. This exploits the existing ACMC structure without controller redesign.

**Fig. 23:**
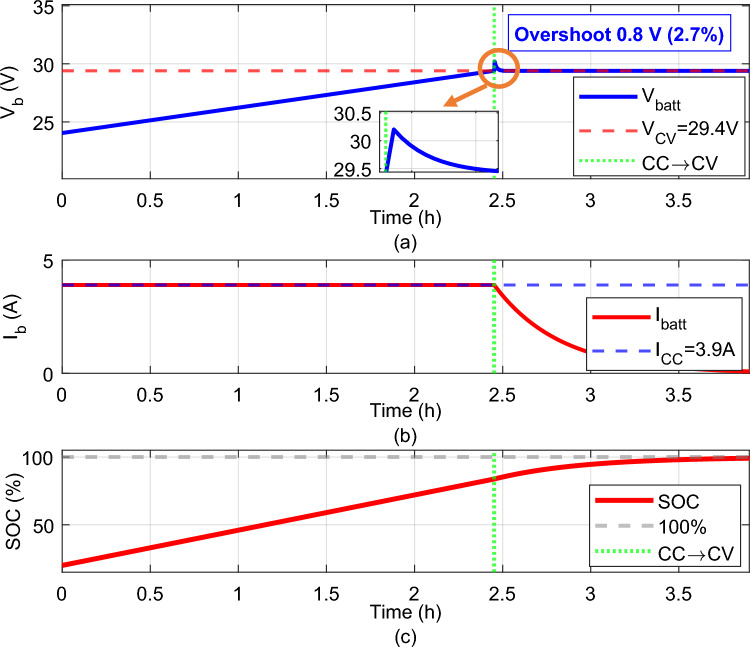
CC-CV charging profile of the ACMC-controlled SIBC (**a**) Battery voltage during CC-CV charging; (**b**) Battery current during CC-CV charging; (**c**) Battery SOC.

Fig. 23 shows the simulated CC-CV profile. During the CC phase, the battery voltage rises linearly from 21 V to 29.4 V while the inductor current remains regulated at 3.9 A. At the CC-CV transition, the voltage overshoot is limited to 0.8 V (2.7%), consistent with the designed 87.4° phase margin. The CV phase exhibits smooth current tapering from 3.9 A to 0.39 A over 1.8 hours, confirming stable voltage-loop regulation. The total charge time of 4.9 hours aligns with standard Level 1 charging expectations for a 20 Ah battery pack. This validation confirms that the proposed ACMC architecture supports direct battery interface without hardware modification, extending the SIBC’s applicability from resistive-load demonstration to practical EV charging scenarios.

## Efficiency, inductor resistance sensitivity analysis, and scalability

### SIBC efficiency evaluation

**Fig. 24:**
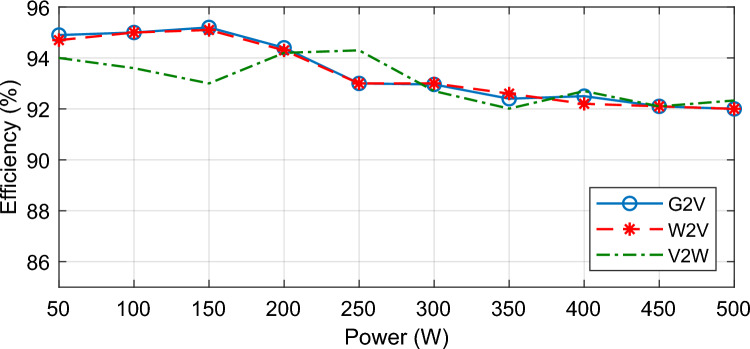
Efficiency Vs Load power.

Fig. 24 presents the measured efficiency of the proposed SIBC across all three operating modes as a function of output power. The converter achieves peak efficiencies of 95.2% in both G2V and W2V modes (modes 1 and 2), while V2W mode (mode 3) reaches a peak of 94.3%. Notably, efficiency remains above 90% in all modes, demonstrating robust performance under varying power demands.

Thermal performance is monitored during continuous operation, with the heat sink temperature stabilizing at 32°C under nominal load. This low thermal stress suggests limited de-rating under tested conditions and confirms that conduction losses rather than switching or core losses dominate at the tested power level. The observed efficiency characteristics align with the non-ideal analysis presented in Section “Non-ideal analysis”, validating the analytical model and establishing confidence for scaling to higher power implementations.

While these efficiency measurements validate SIBC’s performance under nominal conditions, they represent a single point in the design space defined by the specific inductor employed. The 120 mH inductor used in these tests exhibits an equivalent series resistance of 0.15 Ω a value that, per the non-ideal gain model in equations (10-12), introduces measurable voltage drop and conduction loss. Yet the sensitivity of system performance to this parameter remains unquantified. To establish design criteria for inductor selection and to validate the theoretical loss model, the following section examines how variations in inductor resistance influence both conversion efficiency and voltage regulation across the operating range. Table 7 provides loss distribution for each operating mode, including conduction losses, switching losses of the power switches, and losses associated with the inductors.

**Table 7:** Loss Distribution of the proposed SIBC.

Loss distribution	Mode 1 and 2	Mode 3
Switching losses	1.1 W	2.4 W
Conduction losses	2.2 W	3.7 W
Inductor losses	1.15 W	2.87 W
Total losses	4.45 W	8.97 W

###  Detailed loss breakdown and validation

This section provides a detailed breakdown of the total system losses, including semiconductor conduction losses and switching losses, in order to quantify the efficiency limitations of the proposed SIBC. To identify the dominant loss mechanisms and validate the non-ideal model, a component-level loss breakdown is performed for all three operating modes at nominal load. Table 7 presents the measured total losses; this section disaggregates these into semiconductor conduction, switching, and magnetic contributions with explicit calculations.

For the G2V Mode, the conduction loss of the semiconductor is calculated as^27^:

40 $${P}_{c,S1}=D{{I}_{L}}^{2}{R}_{DS(on)}=0.54 W$$

Similarly, for switch S_2,_ the conduction loss is 0.54 W. The diode conduction loss is calculated as follows^27^:

41 $${P}_{c,d2}=(1-D){I}_{L}{V}_{F}=1.29 W$$

Switching losses can be calculated as^27^:

42 $${P}_{s}={f}_{s}({E}_{on}+{E}_{off})=0.87 W$$

The inductor copper losses can be obtained as:

43 $${P}_{cu}={{I}_{L}}^{2}{r}_{L}=2.04 W$$

While the inductor core loss is approximately 0.11W. The total power loss for the G2V mode is obtained as 5.39W, which confirms the measured power loss of 4.45W. Similarly, for V2W mode, the total loss is calculated as 7.58W, which confirms the measured loss of 8.97W. The discrepancy is within measurement uncertainty (±0.5% for voltage, ±1% for current, compounded to ±1.5% power accuracy) and confirms the validity of the non-ideal loss.

###  Inductor series resistance impact on voltage gain

The single-inductor architecture of the proposed SIBC inherently concentrates the dominant magnetic loss mechanisms within a single energy storage element, making overall system performance highly sensitive to the inductor’s equivalent series resistance. Although Section “Efficiency, inductor resistance sensitivity analysis , and scalability” analytically quantified this dependency, the practical design boundaries associated with inductor selection particularly the trade-offs between core characteristics, winding resistance, and achievable efficiency require systematic validation beyond closed-form modeling.

Accordingly, this section investigates the impact of inductor resistance (r_L_) through parametric simulations. The inductor series resistance is intentionally varied within a controlled range to emulate realistic magnetic design scenarios, and the resulting deviations in voltage gain and conversion efficiency are evaluated under steady-state conditions. This simulation-based analysis isolates the r_L_ influence from other parasitic elements, enabling a clear assessment of its direct contribution to gain reduction and efficiency degradation. The results establish the quantitative r_L_ necessary to maintain conversion efficiency above 90% and confirm the accuracy of the non-ideal theoretical model under representative operating conditions.

**Fig. 25:**
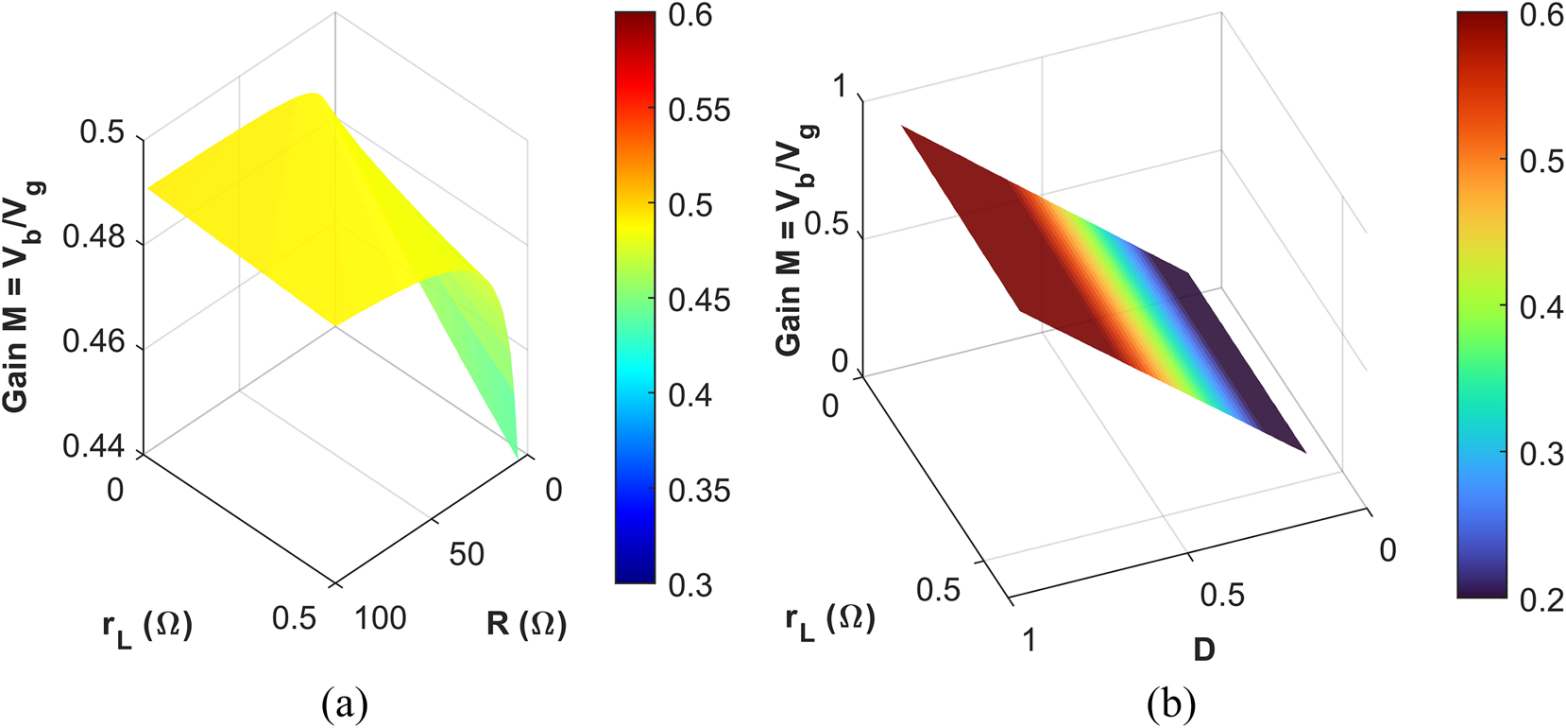
Non-ideal characteristics of the SIBC in G2V Mode (**a**) Gain Vs r_L_ and R-Load at D=0.5; (**b**) Gain Vs r_L_ and D at R=20 Ω.

**Fig. 26:**
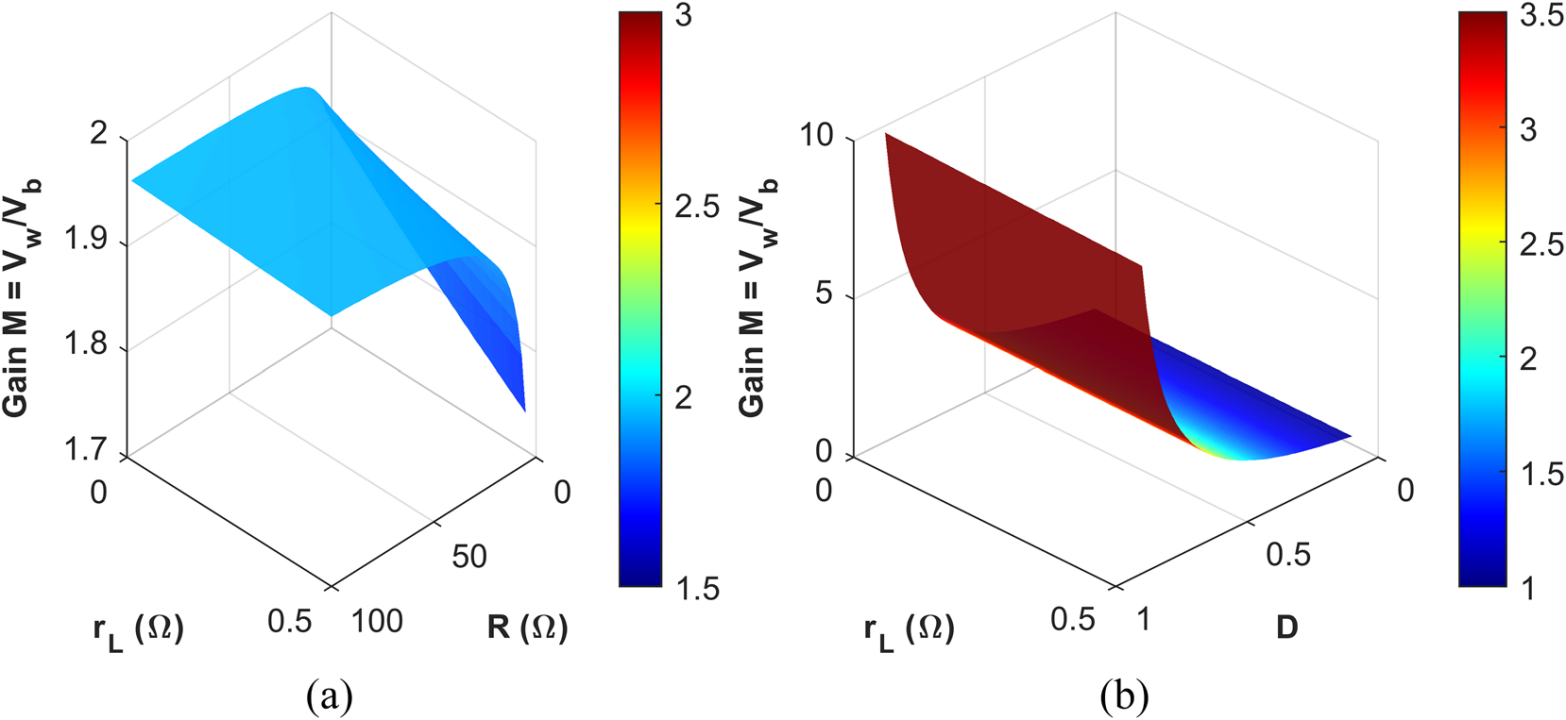
Non-ideal characteristics of the SIBC in V2W Mode (a) Gain Vs r_L_ and R-Load at D=0.5; (**b**) Gain Vs r_L_ and D at R=20 Ω.

**Fig. 27:**
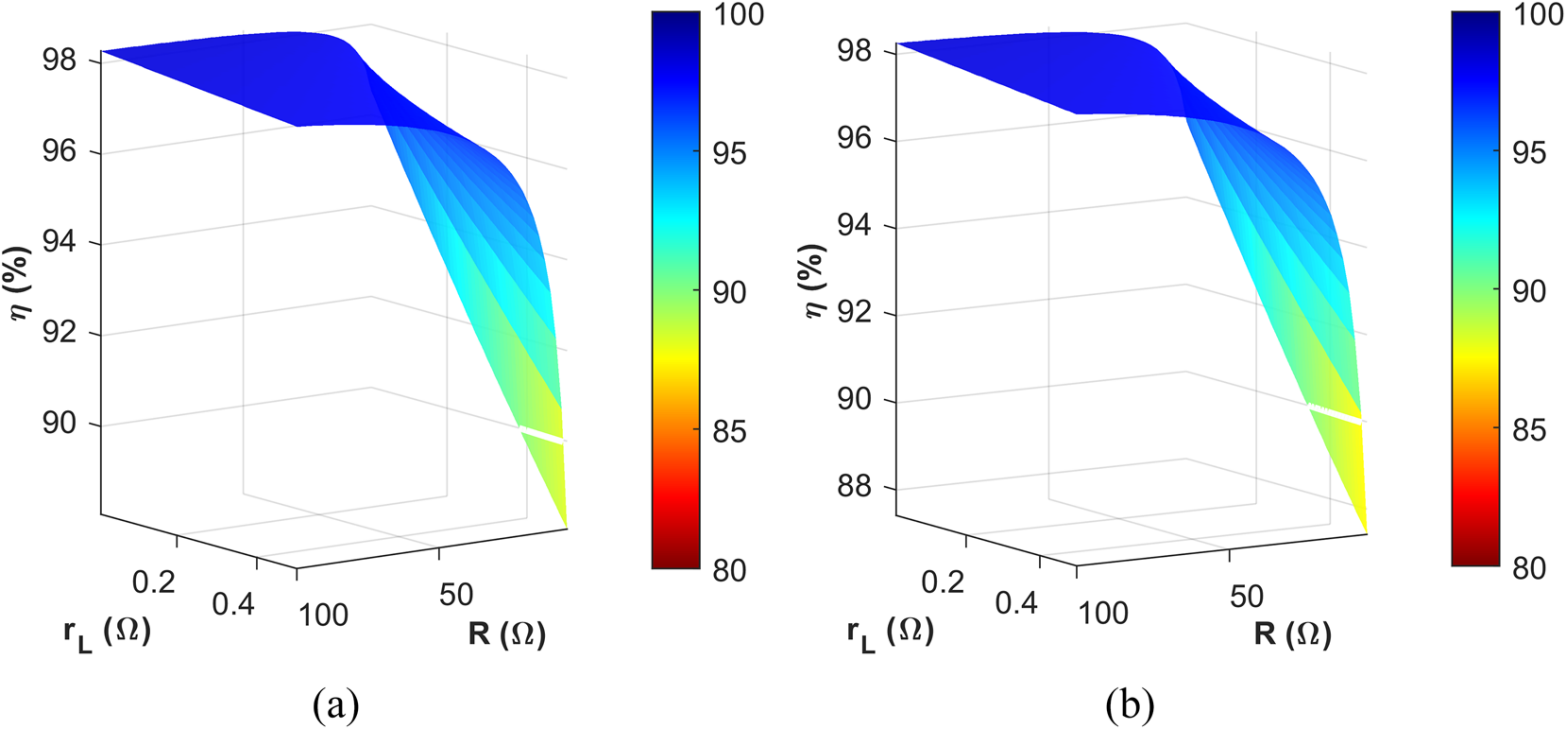
Efficiency Vs r_L_ and R-load at D=0.5 of SIBC (**a**) G2V Mode; (**b**) V2W mode.

Fig. 25 and Fig. 26 present the non-ideal behavior of the SIBC in G2V and V2W modes, emphasizing the influence of r_L_ on voltage gain. As r_L_ increases, both modes exhibit a noticeable reduction in gain and a gradual efficiency drop due to increased conduction losses. This effect becomes more evident at higher duty ratios, where inductor current is larger. The V2W mode shows slightly greater sensitivity, consistent with its higher current stress during boost operation. Overall, the results closely match the derived non-ideal model and clearly demonstrate that minimizing inductor resistance is essential to maintain high efficiency and accurate voltage regulation. The plot of the efficiency varying with r_L_ and the load resistance is shown in Fig. 27 for both modes G2V and V2W.

###  WPT misalignment analysis

**Fig. 28:**
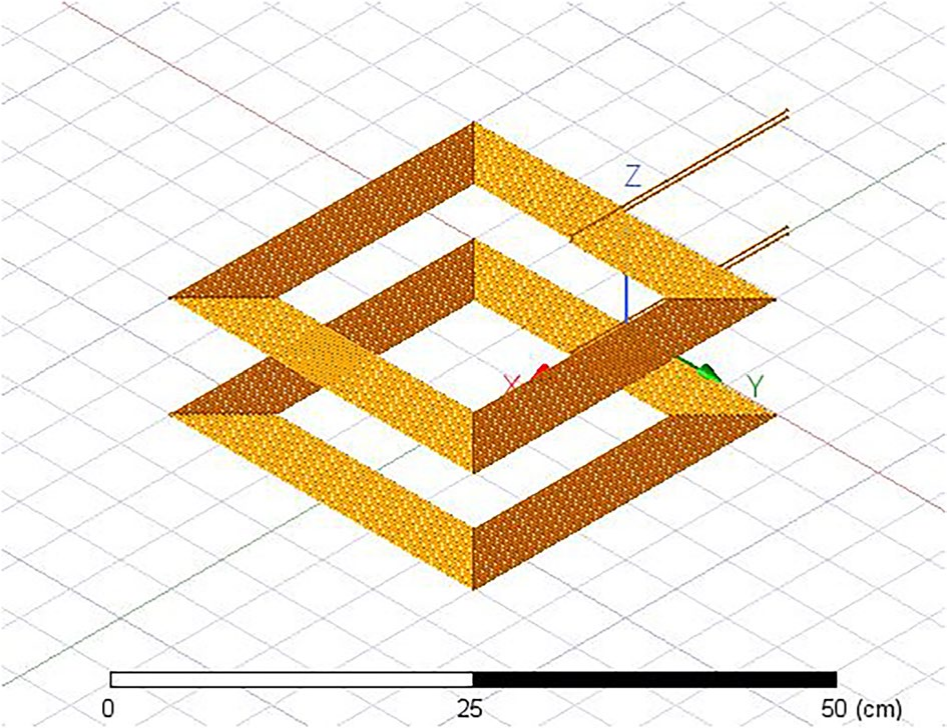
Rectangular coil.

**Fig. 29:**
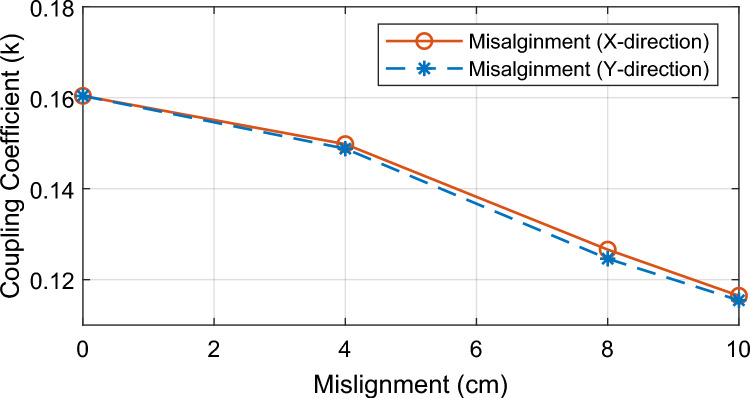
Coupling coefficient (k) Variation with misalignment at an air gap of 10cm.

While experimental validation employs aligned coils (k = 0.16) to isolate converter-level performance, practical V2V charging necessitates tolerance to positional misalignment. To quantify this dependency, a finite-element simulation is conducted in simulation for the 300 mm × 300 mm rectangular coil pair at 10 cm air gap as observed in Fig. 28. Fig. 29 presents the coupling coefficient k versus lateral displacement from 0 to 10 cm. The results confirm that k decays from 0.16 (aligned) to below 0.14 at 6 cm offset, indicating limited misalignment tolerance inherent to conventional rectangular geometries.

The results confirm that the conventional rectangular coil exhibits limited misalignment tolerance compared to bipolar or DD coil configurations, which maintain k > 0.12 under ±10–15 cm displacement^15^. Coil geometry optimization is reserved for future work; the proposed SIBC power stage is agnostic to coil type and will benefit directly from advanced coupling structures without topological modification.

###  Scalability and thermal considerations for 3 kW operation

While the 136 W prototype validates the SIBC operating principles, EV onboard chargers typically require 3–22 kW power levels. Scaling the proposed topology from 136 W to 3 kW introduces thermal and magnetic constraints that must be quantified to establish realistic design boundaries.

####  Semiconductor scaling

At 3 kW and 400 V DC bus voltage, the RMS inductor current increases to I_L_=7.5 A (V2W mode, Vb​=400 V). The IRFP250 MOSFETs used in the prototype are inadequate for 400 V operation and would incur excessive conduction losses. Scaling requires SiC MOSFETs such as the C3M0065090D (900 V, 65 mΩ) or equivalent GaN devices, which provide^28–30^:

- Higher than 2x voltage margin at 400 V bus.
- More than 5x reduction in conduction loss per switch.
- Switching frequency scalability to 100 kHz, reducing inductor volume by 5x.

At 3 kW, total semiconductor conduction losses are estimated to be 14.6 W. Switching losses at 100 kHz with SiC are estimated at 5 W total due to negligible reverse recovery charge^20–22^. The combined semiconductor loss of ≈20 W yields a semiconductor efficiency of 98.3%, compared to ≈95% with Si IGBTs at 20 kHz.

####  Thermal management

Under hood ambient temperatures reach T_a_​=50°C. With SiC device, the junction temperature is limited to T_j,max_​=175°C. The required heat sink thermal resistance is^28,29^:

44 $${R}_{th}<\frac{{T}_{j,max}-{T}_{a}}{{P}_{diss}}-{R}_{th,jc}-{R}_{th,cs}$$

where T_j​_ is the junction temperature, T_a_ is the ambient temperature, T_j,max_ is the maximum junction temperature, R_th,jc_ is the junction-to-case thermal resistance, R_th,cs_ is the case-to-sink thermal resistance, and P_diss_ is the semiconductor power dissipation.

From the above equation, the heat sink thermal resistance is approximately 4.25 K/W. This is readily achievable with a small aluminum extruded heat sink under natural convection, or a significantly smaller profile with forced air. At 6.6 kW (Level 2 OBC), P_diss_​≈45 W and R_th,sa_​<1.8 K/W, requiring forced-air or liquid-cold-plate thermal management.

####  Magnetic scaling

The 120 mH inductor used at 136 W is impractical at 3 kW due to core size and winding resistance. Increasing the switching frequency to 100 kHz reduces the required inductance by the frequency ratio^29,30^:

45 $${L}_{3kW}={L}_{136W}\times \frac{{f}_{s1}}{{f}_{s2}}\times \frac{\Delta {I}_{136W}}{\Delta {I}_{3kW}}\approx 12mH$$

While maintaining the same relative current ripple, a 12 mH inductor on a ferrite core with copper wire winding achieves r_L_​≈0.05Ω.

####  PFC ripple rejection and input filter interaction

The experimental validation employs a regulated 56.5 V DC source to isolate the SIBC performance from front-end interactions. However, in a practical onboard charger, the SIBC is preceded by a single-phase AC-DC PFC stage that produces a DC bus voltage with inherent second-harmonic ripple at twice the line frequency (100 Hz for 50 Hz grids or 120 Hz for 60 Hz grids). This section quantifies the propagation of this low-frequency ripple through the SIBC and validates that the proposed ACMC architecture maintains stable regulation despite the single-inductor structure.

For step-down operation (G2V), the line-to-output voltage transfer function is derived from the small-signal model^22–25^:

46 $${G}_{vg}(s)=D\cdot \frac{1}{1+\frac{s}{Q{\omega}_{o}}+{(\frac{s}{{\omega}_{o}})}^{2}}$$

47 $${\omega}_{o}=\frac{1}{\sqrt{LC}}, Q=R\sqrt{\frac{C}{L}}$$

Substituting L=120 mH, C=220 uF and R=20Ω:

48 $${\omega}_{o}=195 rad/s, Q=0.858$$

At the ripple frequency of 100 Hz, the attenuation is:

49 $$\left|{G}_{vg}(s)\right|\approx 0.054 \left(-25.4 \mathrm{d}\mathrm{B}\right)$$

Thus, a 5% PFC bus ripple (2.8 V peak at 56.5 V) propagates to the battery as only ΔV_b_​≈0.15 V (0.55% of 27.1 V) under open-loop conditions.

The single inductor L provides the primary filtering for the battery current. The impedance of the 120 mH inductor at 100 Hz is Z_L_​=75.4 Ω, which is significantly larger than the battery load resistance (R=20Ω). The resulting battery current ripple is approximately 18.6 mA. This represents only 0.5% of the 3.69 A average battery current and is well within acceptable limits for Li-ion battery charging (typically 5% current ripple).

Under closed-loop ACMC operation, the outer voltage loop bandwidth of 300 Hz provides additional ripple rejection. The loop gain at 100 Hz is approximately 10 dB as observed from Fig. 18, yielding an output impedance reduction factor of 3.16. The resulting closed-loop ripple attenuation exceeds 35 dB, limiting the battery voltage ripple to 50 mV (0.2%) and the current ripple to less than 6 mA.

While the single inductor and 220 uF capacitor provide sufficient 100 Hz attenuation for the 136 W prototype, scaling to 3 kW reveals a constraint. The output capacitor must absorb the ripple power, causing a voltage variation:

50 $$\Delta {V}_{g}=\frac{{P}_{o}}{2\omega {V}_{g}C}$$

At 3 kW, 400 V bus, and 100 Hz, maintaining ΔV_g_​<10 V (2.5%) requires a capacitor larger than 597 uF. Therefore, a practical 3 kW OBC requires a capacitor larger than 1000 uF on the DC bus or a dedicated LC pre-filter stage between the PFC and SIBC to prevent excessive bus voltage drop. This pre-filter does not compromise the SIBC’s single-inductor architecture, as it is shared with the PFC stage and does not participate in the bidirectional energy transfer control.

**Fig. 30:**
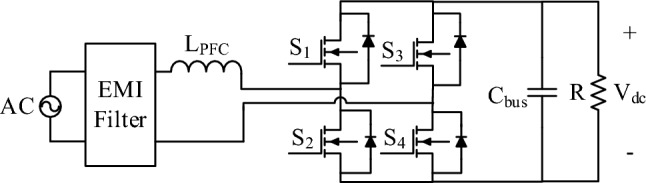
Bridgeless totem pole PFC circuit diagram.

A standard bridgeless totem-pole PFC or boost PFC converter is typically employed for single-phase OBC applications^1,2,28^. These topologies achieve power factors >0.99 and THD <5% at rated power, satisfying IEC 61000-3-2 Class A limits for harmonic current emissions. The PFC output is a regulated DC bus (400 V for Level 2 OBC) with inherent second-harmonic ripple at 100 Hz (50 Hz grid) or 120 Hz (60 Hz grid). The bridgeless totem-pole PFC used in simulations is shown in Fig. 30^31^.

The following calculations are used for the design of the bridgeless totem-pole PFC converter^31^. The instantaneous grid voltage is:

51 $${v}_{ac}\left(t\right)=325.27\mathrm{s}\mathrm{i}\mathrm{n}(2\pi \cdot 50\cdot t)$$

The duty cycle of the slow-leg commutating switch varies as:

52 $${D}_{PFC}\left(t\right)=1-\frac{\left|{v}_{ac}\left(t\right)\right|}{{V}_{bus}}$$

The PFC boost inductor is sized for 20 % current ripple at peak input voltage. The peak input current is:

53 $${I}_{P}=\frac{\sqrt{2}{P}_{o}}{{v}_{ac,rms}\cdot PF}\approx 18.5 A$$

where P_o_ is the output power, PF is the power factor.

With current ripple of 20% the inductance is calculated as:

54 $${L}_{PFC}=\frac{{v}_{p}\times {D}_{PFC,min}}{\Delta {I}_{L}\times {f}_{s}}\approx 164\mu H$$

where v_p_ is the peak voltage, ∆I_L_ is the current ripple. A standard 180 µH ferrite-core inductor is selected. The DC-link capacitor must limit second-harmonic (100 Hz) voltage ripple to less than 10 V (2.5 % of 400 V). From the ripple power balance:

55 $${C}_{bus}\ge \frac{{P}_{o}}{2{\omega}_{l}\times {v}_{bus}\times \Delta {v}_{bus}}=\frac{3000}{2\times 2\pi \times 50\times 400\times 10}\approx 1194\mu F$$

Where ω_l_ is the line angular frequency , ∆v_bus_ is the DC bus voltage ripple.

The PFC employs ACMC, consistent with the ACMC architecture proposed for the SIBC. A multiplier generates a sinusoidal current reference i_ref_​(t) proportional to the rectified grid voltage. The inner current loop (crossover ≈ 10 kHz) forces the inductor current to track i_ref_​(t), while the outer voltage loop (crossover ≈ 10 Hz) regulates V_bus_​. This dual-loop structure naturally yields near-unity power factor because the current reference is synchronized to the grid voltage. The control law for the current compensator G_ci,PFC_​(s) is a Type II compensator:

56 $${\mathrm{G}}_{\mathrm{c}\mathrm{i},\mathrm{P}\mathrm{F}\mathrm{C}} \left(\mathrm{s}\right)={\mathrm{K}}_{\mathrm{c}\mathrm{i}}\frac{1+\frac{{\omega}_{zi}}{s}}{1+\frac{s}{{\omega}_{pi}}}$$

Where with K_ci​_=8.2 , zero at 1.5 kHz, and pole at 50 kHz, yielding 65° phase margin at 10 kHz.

**Fig. 31:**
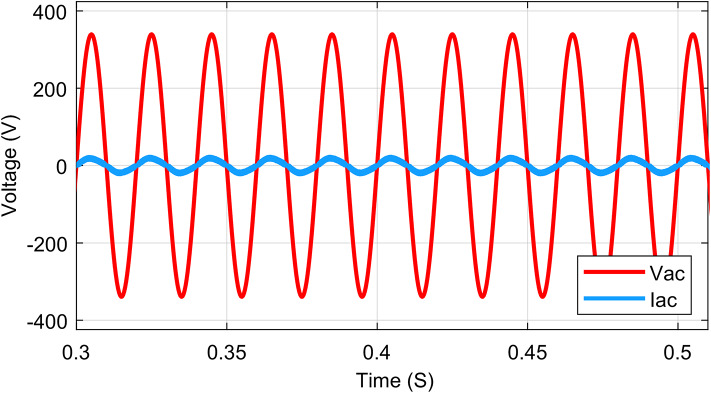
Grid voltage & current waveforms at 3 kW.

To validate harmonic compliance, the grid current i_ac_​(t) is simulated under full-load (3 kW). Fig. 31 presents the steady-state waveforms, confirming that i_ac_​(t) is sinusoidal and in phase with v_ac_​(t).

**Fig. 32:**
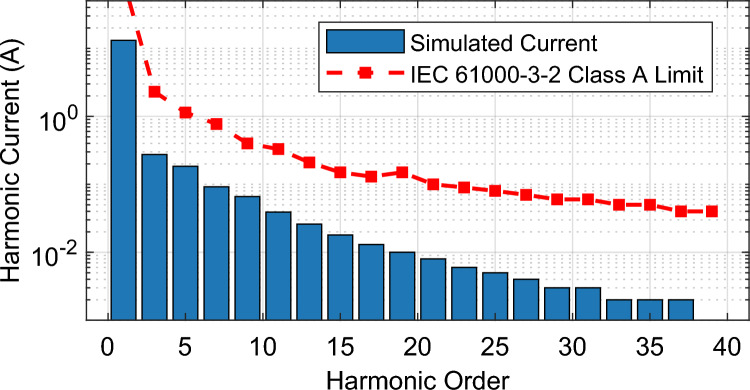
Harmonic spectrum vs. IEC 61000-3-2 class A limits.

**Fig. 33:**
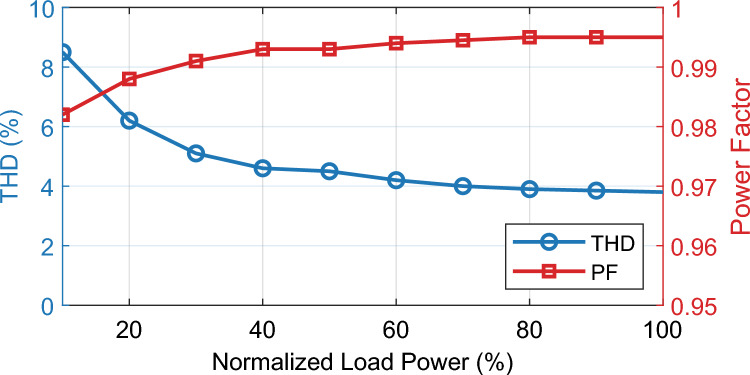
THD and power factor vs. normalized load power.

Fig. 32 plots the harmonic spectrum of the grid current at 3 kW. All individual odd harmonics are below the regulatory thresholds, with the third harmonic at 2.1 % of fundamental and the fifth at 1.4 %. Fig. 33 presents the THD and PF as a function of normalized output power. THD remains below 5 % from 30 % to 100 % load, and PF exceeds 0.99 above 30 % load, confirming robust performance across the EV charging profile.

##  Conclusion

This paper proposed a Single Inductor Bidirectional Converter (SIBC) that unifies grid, battery, and wireless power transfer within a single three-port power stage, eliminating cascaded structures and mode-switching relays. The main novelty lies in achieving native bidirectional wired (G2V) and wireless (V2W, and W2V) operations through unified modulation of only four switches and one inductor, without hardware reconfiguration. A non-ideal steady-state analysis quantified inductor ESR boundaries for sustaining >90 % efficiency, while a complete small-signal model identified a boost-type RHP zero in V2W mode; the proposed two-loop ACMC mitigated this limitation, achieving 40× bandwidth improvement and 3.8 ms settling time. Experimental validation on a 136 W prototype demonstrated DC–DC efficiencies of 93.4 % (transmitter) and 95.07 % (receiver), and 75.03 % end-to-end efficiency, while a 1 kW interim hardware test and simulation confirmed CC-CV battery charging compatibility, front-end PFC integration achieving THD < 4 % and PF > 0.99, and scalable operation toward commercial Level 2 onboard charger deployment. Future work will employ advanced coil geometries to improve misalignment tolerance, validate a 3 kW SiC-based closed-loop prototype, and extend the control framework to Vehicle-to-Grid operation.

## Data Availability

The data generated during and/or analysed during the current study are available from the corresponding author on reasonable request.
